# Development Status of Fe-V-Based High-Entropy Alloys for Nuclear Reactors

**DOI:** 10.3390/ma19183913

**Published:** 2026-09-15

**Authors:** Haotian Wu, Yuming Fu, Pingping Liu, Tonghao Wang, Wentuo Han, Xiaoou Yi, Qian Zhan, Farong Wan

**Affiliations:** School of Materials Science and Engineering, University of Science and Technology Beijing, Beijing 100083, China; whtustb@163.com (H.W.); kindred396@163.com (Y.F.); wthustb@163.com (T.W.); han-wt@ustb.edu.cn (W.H.); xiaoouyi@ustb.edu.cn (X.Y.); qzhan@mater.ustb.edu.cn (Q.Z.); wanfr@mater.ustb.edu.cn (F.W.)

**Keywords:** Fe-V-based high-entropy alloys, low activation, calculation-driven design, irradiation damage, nuclear energy

## Abstract

**Highlights:**

• The application of the multiscale computational framework has significantly reduced the development costs of high-entropy alloys.
• The development of bimetallic alloys will help solve the problem of alloy brittleness.
• The advancement of preparation techniques will drive the enhancement of alloy performance.
• The synergistic effect of H and He, along with the irradiation damage behavior of the alloys under high dpa, still requires in-depth research.

**Abstract:**

Against the backdrop of the “dual carbon” strategy, clean and efficient energy is the goal that people pursue. Nuclear fusion energy is recognized as a clean energy source worldwide. Due to the high-energy neutrons produced during nuclear fusion reactions, the materials for nuclear fusion reactors are required to be low in activation while also having high resistance to irradiation damage. Both Fe and V are completely low-activation elements, and high-entropy alloys currently exhibit excellent radiation resistance properties. Therefore, Fe-V-based high-entropy alloys may become a competitive candidate material for future fusion reactors, although their performance under neutron irradiation and long-term high-temperature service remains to be validated. This paper systematically reviews the research progress in the design methods, preparation methods, mechanical properties, thermal stability, and radiation resistance of Fe-V-based high-entropy alloys. In response to the high strength and low activation requirements of structural materials for fusion reactors, a development strategy of Fe-V-based high-entropy alloys using FeCrV-based alloys as the main component and supplemented by low-activation elements such as Mn and Ti is proposed. This provides certain references for the development and design of new-generation reactor structural materials.

## 1. Introduction

With the continuous growth of global energy demand and the advancement of carbon neutrality goals, nuclear energy has become increasingly important as a clean energy source. Fission reactors and fusion reactors, as two feasible ways to utilize nuclear energy at present, have achieved extensive research results. Generation IV nuclear reactors possess advantages such as high efficiency, economy, safety, and sustainability [1]. However, the service environment of Generation IV nuclear reactors poses more severe challenges to their structural materials. For example, components in gas-cooled fast reactors will withstand pressures up to 5–8 MPa, operating temperatures of 800 °C, and extreme fast neutron irradiation [2]. The operating temperature of very-high-temperature gas-cooled reactors will even exceed 1000 °C, and the ultra-high temperature will inevitably reduce the service life of materials. Lead-cooled fast reactors (LFRs), molten salt reactors (MSRs), and sodium-cooled fast reactors (SFRs) use liquid metals or molten salts as coolants, with relatively low high-temperature mechanical loads, but usually result in higher damage levels of structural materials and more severe corrosion reactions. Among them, LFRs use heavy metals as coolants, which have the advantage of high heat capacity and can provide significant thermal inertia in the event of an accident to improve safety. However, lead (Pb) and bismuth (Bi) exhibit strong chemical corrosion under high temperature and irradiation [3]; fluoride salts commonly used in MSRs also have strong corrosivity, and fluoride salts can dissolve metal oxides, making the surface of structural metals unable to form passivating oxide films and more susceptible to corrosion. Supercritical water-cooled reactors (SCWRs) face the problem of stress corrosion cracking [4]. The operating environment of fusion reactors is even more extreme: the neutron energy generated by fusion reactions is as high as 14.1 MeV, while the neutron energy generated by fission reactions is only about 1 MeV. High neutron energy not only leads to a significant increase in displacement damage levels but also induces nuclear transmutation [5]. Compared with the current generation, structural materials for Generation IV reactors and fusion reactors generally face stronger irradiation damage, corrosive environments, and high-temperature mechanical loads. This requires materials to have excellent corrosion resistance, high-temperature resistance, irradiation resistance, low neutron activation, and high mechanical strength to further improve economy, safety, and sustainability [2,3].

Among the advanced reactor materials currently under development, austenitic stainless steel has a working temperature of only 300–400 °C and is relatively prone to swelling and embrittlement under neutron irradiation [6], nickel-based alloys have excellent high-temperature performance (e.g., GH3535 alloy), but suffer from aggravated helium damage due to nuclear transmutation reactions [7], tungsten and tungsten-based alloys have high brittleness and thermal expansion coefficients, leading to interface cracking and fatigue failure [4], including nanometals and nanoceramics, face the problem of thermal stability of their microstructures [8], low-activation ferritic–martensitic steels have an intractable problem of irradiation-induced catalysis [9]. To meet the service requirements of new-generation advanced nuclear reactors, the development of new nuclear materials with excellent comprehensive performance has become the key to advancing nuclear technology.

High-entropy alloys (HEAs) are a new type of multi-principal element solid solution alloys. The concept was first proposed by Yeh [10] and Cantor [11] et al. in 2004, changing the traditional alloy design idea that focuses on one or a few elements. HEAs are usually composed of multiple principal elements with equal atomic ratios, where each element mainly exists in the form of a single solid solution or a small amount of precipitates, and there is no distinction between solute and solvent among elements. HEAs have characteristics such as high mixing entropy effect, lattice distortion effect, sluggish diffusion effect, and cocktail effect [12]. Their compositions are adjustable, enabling them to combine the advantages of multiple principal elements and overcome the shortcomings of single-principal element alloys, thus exhibiting excellent mechanical, thermal, and irradiation-resistant properties.

At present, HEAs have received extensive research. Refractory high-entropy alloys (RHEAs) containing high-melting-point transition metal elements including Nb, Ta, Cr, Mo, W, Re, Zr, and Hf have melting points above 2000 K and corrosion resistance, making them important candidate materials for Generation IV nuclear reactors such as LFRs and MSRs [13]; studies have found that aluminum-based alloys reinforced with high-entropy fibers can retain the excellent irradiation resistance of HEAs while significantly reducing the weight of nuclear reactor structural materials, thereby lowering maintenance costs and promoting modular construction [14]; Ta-Ti-V-Zr-X low-activation HEAs have lower induced radioactivity compared with traditional stainless steel materials, and their waste materials cause less pollution to the environment, meeting the requirements of clean and sustainable development of nuclear energy [15].

Avoiding the formation of intermetallic compounds and obtaining alloys with a single solid solution phase is an important consideration in HEA design. Therefore, the theory of “like dissolves like” has an important influence on HEA developers. When selecting constituent elements for most HEA designs, elements with the same crystal structure and atomic radius are often chosen. Elements in the fourth period of the periodic table, such as Ti, V, Cr, Mn, and Fe, not only have certain similarities in geometric topological structure and electronegativity but also exhibit low activation characteristics in nuclear material applications. Among them, Fe-based HEAs have always been the focus of research due to their economy and comprehensive performance. V has a high melting point, and adding it to HEAs can improve high-temperature performance. Fe-V-based HEAs show broad application prospects in structural materials for Generation IV nuclear reactors. Yin et al. [16] found that due to its special atomic mismatch volume, V can induce lattice distortion in both FCC-phase Co-Cr-Fe-Mn-Ni-V and BCC-phase Cr-Mo-Nb-Ta-V-W-Hf-Ti-Zr HEA families, thereby improving the mechanical strength of the alloys. Li et al. [17] added Mn, Ti, and V in equimolar ratios to the AlCrFeCoNiCu alloy, and their experimental results showed that adding V to the AlCrFeCoNiCu alloy had the best strengthening effect and the smallest decrease in ultimate strain. Zhang et al. [18] studied the irradiation performance of V_x_CrFeMnNi_y_ (x = 0, y = 2; x = 1, y = 0) HEAs, and the results showed that the presence of V inhibits He bubble growth and irradiation hardening. It is worth noting that V can lead to the formation of brittle σ phases, thereby reducing the plasticity, toughness, and corrosion resistance of the alloy, which is the reason why traditional V-based alloys have problems such as low-temperature irradiation embrittlement, poor high-temperature strength, and creep resistance. By adding different elements, adjusting the content of each element, and adopting different preparation methods, it is expected to design Fe-V-based HEAs with excellent high-temperature performance. Ma et al. [19] designed and prepared a series of V_x_Fe_65−x_Cr_15_Mn_20_ HEAs, whose yield strength is as high as 520 MPa at 800 °C and 275 MPa at 900 °C, higher than conventional low-activation steels and V-based alloys. Jo et al. [20] successfully improved the low-temperature strength by utilizing room-temperature deformation twinning in partially recrystallized VCrMnFeCoNi HEAs through thermodynamic calculations.

**Figure 1:**
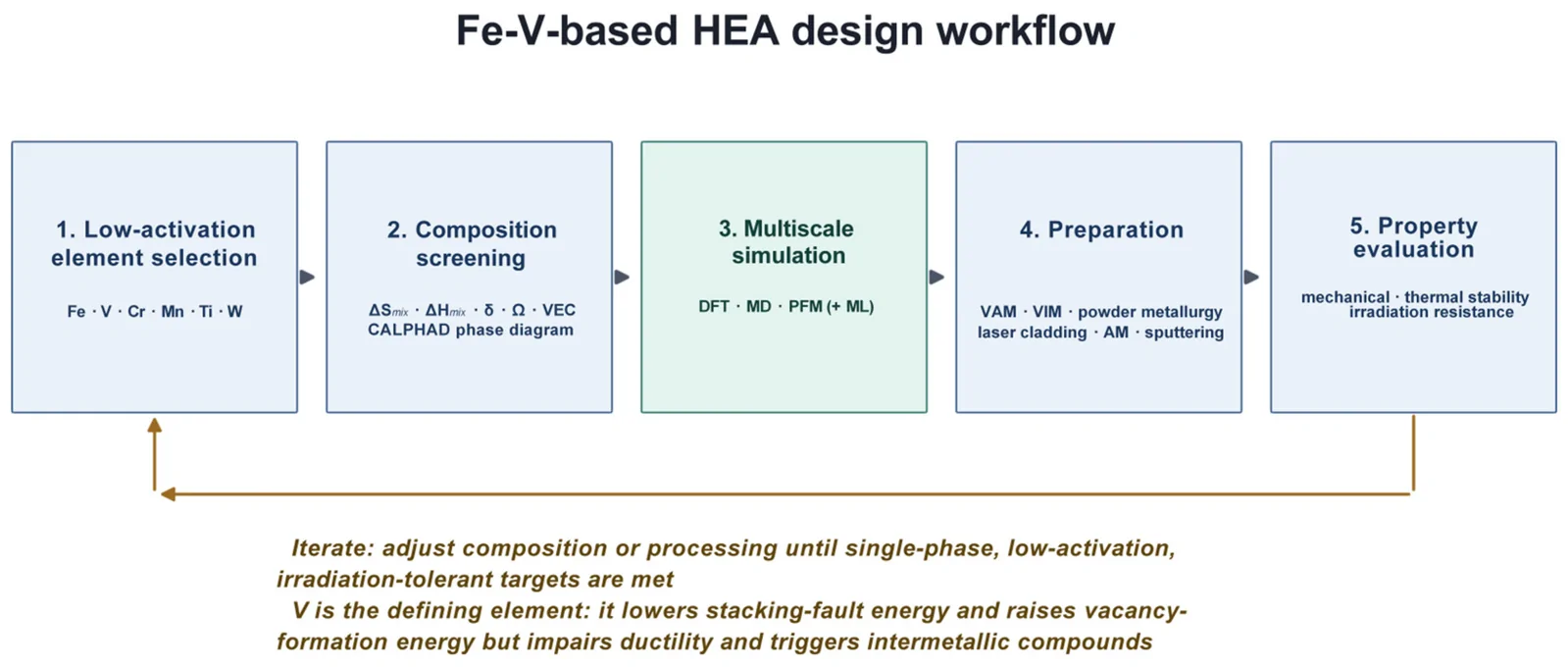
Schematic of the Fe-V-based HEA design workflow, from low-activation element selection and empirical plus CALPHAD screening, through multiscale simulation (DFT, MD, PFM, ML) and preparation, to property evaluation and iteration.

Significant progress has been made in the development of Fe-V-based HEAs, but most existing systems contain non-low-activation elements such as Ni and Co, limiting their application in nuclear reactors. The compositional adjustability of HEAs brings broad research prospects for their development. In recent years, numerous advances have been made in the design, preparation, and performance research of HEAs. In particular, the combination of machine learning and artificial intelligence with conventional simulation and calculation methods has greatly reduced the development cost of HEAs and strongly promoted research on the reaction mechanisms and regulatory factors of HEAs [21]. This paper systematically reviews the research progress of Fe-V-based HEAs in terms of design methods, preparation processes, mechanical properties, thermal stability, and irradiation resistance, aiming to provide a reference for the research and development of this series of alloys and their application in the nuclear energy field. The design workflow of HEAs is shown in Figure 1. By comprehensively sorting out existing achievements, we hope to open up new paths for the application of HEAs in key fields such as nuclear energy.

### 1.1. Review Methodology

This review focuses on Fe-V-based high-entropy alloys (HEAs) and their closely related medium-entropy and multi-principal-element counterparts intended for low-activation structural applications in advanced fission and fusion reactors. We adopt the following convention: high-entropy alloys (HEAs) denote alloys with five or more principal elements, medium-entropy alloys (MEAs) denote alloys with three or four principal elements, and multi-principal-element alloys (MPEAs) serve as the general umbrella covering both. The Fe-V-based family reviewed here is centred on Fe-Cr-V and related low-activation systems, in which V is the defining addition that distinguishes these alloys from conventional Fe-Cr-based steels. Alloys containing non-low-activation elements such as Ni and Co are discussed only where they inform the design principles or the irradiation behaviour of the low-activation Fe-V family.

### 1.2. Scope of Reviewed Materials

The literature was compiled by searching the Web of Science, Scopus and Google Scholar databases using combinations of the keywords “Fe-V high-entropy alloy”, “FeCrV”, “V-containing high-entropy alloy”, “low-activation high-entropy alloy”, “medium-entropy alloy” and “multi-principal-element alloy”, each combined with “nuclear”, “irradiation”, “mechanical properties”, “thermal stability”, “composition design” and “preparation”. The search covered publications from 2004 to 2026, and the reference lists of retrieved reviews were screened for additional primary studies. Records were screened by title and abstract, and the full texts were assessed for relevance to composition design, simulation, preparation, mechanical properties, thermal stability or irradiation behaviour of Fe-V-containing alloys.

### 1.3. Scientific Contribution

A recent review by Xu et al. [22] comprehensively summarized FeCrV-based multi-component alloys for advanced nuclear energy applications, with emphasis on the FeCrV family, its preparation and its mechanical and irradiation properties. The present review differs in scope and contribution in four respects. First, it adopts an explicitly low-activation, Fe-V-centric scope oriented toward fusion and Generation IV applications. Second, it devotes a dedicated section to composition-design criteria and to the four multiscale simulation routes (DFT, CALPHAD, MD and PFM), with an explicit comparison of their strengths, limitations and applicability to Fe-V systems. Third, it organizes the irradiation section by crystal structure (FCC versus BCC) and synthesizes the underlying defect and helium-bubble mechanisms, including a comparison with RAFM steels and vanadium alloys. Fourth, it provides a forward-looking roadmap that integrates machine learning, high-throughput experimentation and multiscale modelling.

## 2. Composition Design

### 2.1. Theoretical Basis for HEA Composition Design

The excellent mechanical properties of HEAs are inseparable from the solid solution strengthening effect of the alloys. Therefore, inferring whether an alloy can form a single solid solution is the basis of HEA composition design. Through the analysis of the atomic radius, crystal structure, electronegativity of each element, and thermodynamic parameters of the alloy system, a variety of empirical criteria have been developed to infer the phase composition of the selected HEA system.

Zhang et al. [23] modified and optimized the Hume-Rothery criteria to predict new high-entropy alloy systems based on experimental data and statistical analysis of the results. They proposed semi-empirical criteria for the relationship between mixing entropy (${∆S}_{\mathrm{mix}}$), mixing enthalpy (${∆H}_{\mathrm{mix}}$) and atomic radius difference (δ) with solid solution formation:

(1) $$\begin{array}{c} {∆S}_{mix}=-R\sum_{i=1}^{n}C_{i}{lnC}_{i} \end{array}$$

(2) $$\begin{array}{c} {∆H}_{mix}=\sum_{i=1}^{n}{4∆H}_{ij}^{mix}C_{i}C_{j} \end{array}$$

(3) $$\begin{array}{c} \delta =\sqrt{\sum_{i=1}^{n}C_{i}{\left(1-r_{i}/\bar{r}\right)}^{2}} \end{array}$$

where $C_{i}$ and $C_{j}$ are the atomic percentages of the *i*-th and *j*-th elements, respectively; *n* is the number of alloy components; ${∆H}_{\mathrm{ij}}^{\mathrm{mix}}$ represents the mixing enthalpy of the *i*-th and *j*-th elements obtained from the Miedema model [24]. R is the gas constant; $r_{i}$ is the atomic radius of the i-th element; $\bar{r}=\sum_{i=1}^{n}C_{i}r_{i}$ is the average atomic radius of the system.

When the mixing enthalpy (${∆H}_{\mathrm{mix}}$) approaches zero, the alloy system is more inclined to form a single phase. Therefore, to obtain a single solid solution, the critical range of mixing enthalpy is generally −15 kJ·mol^−1^ < ${∆H}_{\mathrm{mix}}$ < 5 kJ·mol^−1^ [25]. In addition, according to the research of Mizutani [26], a solid solution phase can only be formed when the atomic size difference *δ* between solute and solvent elements is less than 6.6%.

It is obviously insufficient to infer the phase composition of alloys only through the above criteria. Further research and statistics have found that electronegativity difference (Δχ) and average valence electron concentration (*VEC*) play an important role in inferring the type of phase formed by the alloy [27,28,29]. Their definitions are as follows:

(4) $$\begin{array}{c} VEC=\sum_{i=1}^{n}C_{i}{\left(VEC\right)}_{i} \end{array}$$

(5) $$\begin{array}{c} ∆\chi =\sum_{i=1}^{n}C_{i}\left(1-\frac{\chi_{i}}{\bar{\chi }}\right) \end{array}$$

where ${\left(\mathrm{VEC}\right)}_{i}$ is the valence electron concentration of the *i*-th element; $\chi_{i}$ is the electronegativity of the *i*-th element; $\bar{\chi }$ is the molar average electronegativity.

According to the research results of Guo et al. [30], *VEC* < 6.87 is conducive to the formation of the BCC phase, *VEC* ≥ 8 results in a stable FCC phase, and 6.87 ≤ *VEC* < 8 leads to the coexistence of FCC and BCC phases. According to the Hume-Rothery rule, a small difference in electronegativity is conducive to the formation of solid solutions. Although ∆χ can often not be used alone to predict phase formation, its value is expected to provide important clues about the formation of alloy phase composition [31].

To explore how to easily obtain simple solid solution phases, Yang et al. [25] found that $\left|H_{mix}\right|$ and $T_{m}\cdot {\Delta S}_{mix}$ play completely different roles in the formation of solid solutions. Based on the experimental results of Miedema [32], they inferred that the larger the absolute value of $H_{mix}$, the more difficult it is to form a solid solution phase. Only when the value of $H_{mix}$ is close to zero can different elements be randomly distributed in the alloy, and the solid solution phase can stably appear in the solid phase. Therefore, the absolute value of mixing enthalpy $\left|H_{mix}\right|$ is used to describe the resistance to solid solution formation. In addition, since ${\Delta S}_{mix}$ of multi-component alloys is always positive, it can be regarded as the driving force for solid solution phase formation in Gibbs free energy. Based on this result, a new parameter Ω is defined:

(6) $$\begin{array}{c} \Omega =\frac{T_{m}{\Delta S}_{mix}}{\left|{\Delta H}_{mix}\right|} \end{array}$$

where $T_{m}$ is the predicted melting point of the multi-component alloy, which can be approximately calculated as

(7) $$\begin{array}{c} T_{m}=\sum_{i=i}^{n}C_{i}{\left(T_{m}\right)}_{i} \end{array}$$

${\left(T_{m}\right)}_{i}$ is the melting point of each element.

Yang considered that when Ω is greater than 1.1, simple solid solutions are more likely to form in HEAs.

Similarly, following the same idea, Singh [33] et al. established a new parameter Λ from the geometric parameters of the material structure and defined it as

(8) $$\begin{array}{c} \Lambda =\frac{{\Delta S}_{mix}}{\delta^{2}} \end{array}$$

where δ is the atomic radius difference mentioned above. In this formula, δ is concretely expressed as a measure of the strain of the distorted lattice of the alloy relative to the perfect lattice. Therefore, δ is also regarded by them as the resistance to the formation of solid solution phases in HEAs.

Singh considered that when Λ < 0.24, intermetallic compounds will form in the alloy; when 0.24 < Λ < 0.96, the alloy will exhibit two phases; when 0.96 < Λ, the alloy is more inclined to form a single solid solution phase.

Wang et al. [34] also started from the geometric structure and proposed a parameter *γ* that can reflect the difference between the maximum and minimum atomic radii. Through statistics, it was found that *γ* < 1.175 is a necessary condition for forming a solid solution. *γ* is expressed as

(9) $$\begin{array}{c} \gamma =\left(1-\sqrt{\frac{{\left(r_{s}+\bar{r}\right)}^{2}-{\bar{r}}^{2}}{{\left(r_{s}+\bar{r}\right)}^{2}}}\right)/\left(1-\sqrt{\frac{{\left(r_{L}+\bar{r}\right)}^{2}-{\bar{r}}^{2}}{{\left(r_{L}+\bar{r}\right)}^{2}}}\right) \end{array}$$

where $r_{L}$ and $r_{s}$ are the maximum and minimum atomic radii in the alloy, respectively.

In addition, Ghosh et al. [35] re-examined the concept of configurational entropy of HEAs and considered that the traditional configurational entropy, which considers the alloy as an ideal gas, lacks consideration of the size and packing mode of constituent elements. Through statistics and research, a new parameter ϕ is defined:

(10) $$\begin{array}{c} \phi =\frac{{\Delta S}_{mix}-\frac{\left|{∆H}_{mix}\right|}{T_{m}}}{\left|{\Delta S}_{E}\right|} \end{array}$$

The total configurational entropy of alloy mixing ${\Delta S}_{T}$ can be expressed as ${\Delta S}_{T}$ = ${\Delta S}_{mix}$ + ${\Delta S}_{E}$, where ${\Delta S}_{mix}$ is the configurational entropy of ideal gas mixing, and ${\Delta S}_{E}$ is the excess entropy of mixing, which is a function of atomic packing and atomic size. According to the research of their team [36], the magnitude of ${\Delta S}_{E}$ is closely related to the atomic size mismatch of a given atomic filler. When ϕ > 20, the alloy will form a single-phase solid solution.

The above criteria all aim to reflect the tendency to form solid solution phases. The parameters of these criteria are usually determined by outlining the regions related to solid solution phase formation from existing alloy diagrams. Although the parameters in these criteria often reflect some energetic elements of the alloy, the models do not explicitly rely on the free energy of the solid solution phase to predict the phase type. Instead, the phase type is mainly determined by the topological or chemical similarity of the constituent atoms. Therefore, the above models can be regarded as a continuation of the Hume-Rothery rule to a certain extent. Topological similarity is usually represented by changes in atomic size between constituent elements (such as δ, γ, Λ, and ${\Delta S}_{E}$ parameters), while chemical similarity is usually represented by the mixing enthalpy (${∆H}_{mix}$) between constituent elements. The more similar the alloy components are, the greater the tendency to produce solid solution phases.

Table 1 summarizes the atomic radius (*r*), melting point (*T*_m_), density (*φ*_0_), valence electron concentration (*VEC*), electronegativity (*χ*), room-temperature crystal structure, and high-temperature crystal structure of common elements in HEAs.

**Table 1:** Structure and some physical properties of commonly used high-entropy alloy elements [37].

Element	r/Å	T_m_/K	ρ_0_/(g/cm^3^)	VEC	χ	RT Structure	T_m_ Structure
Be	1.128	1560	1.86	2	1.57	HCP	BCC
B	0.820	2348	2.46	3	2.04	hR105	hR105
C	0.773	4742	2.27	4	2.55	HCP	HCP
N	0.750	63	1.03	5	3.04	-	-
O	0.730	54	0.92	6	3.44	-	-
Al	1.432	933	2.70	3	1.61	FCC	FCC
Si	1.153	1687	2.33	4	1.90	cubic	cubic
Sc	1.641	1814	3.00	3	1.36	HCP	BCC
Ti	1.462	1941	4.50	4	1.54	HCP	BCC
V	1.316	2183	6.12	5	1.63	BCC	BCC
Cr	1.249	2180	7.19	6	1.66	BCC	BCC
Mn	1.350	1519	7.47	7	1.55	cl58	BCC
Fe	1.241	1811	7.88	8	1.83	BCC	BCC
Co	1.251	1768	8.84	9	1.88	HCP	FCC
Ni	1.246	1728	8.91	10	1.91	FCC	FCC
Cu	1.278	1358	8.94	11	1.90	FCC	FCC
Zn	1.395	693	7.14	12	1.65	HCP	HCP
Y	1.802	1795	4.47	3	1.22	HCP	BCC
Zr	1.603	2128	6.51	4	1.33	HCP	BCC
Nb	1.429	2750	8.58	5	1.60	BCC	BCC
Mo	1.363	2896	10.23	6	2.16	BCC	BCC
Hf	1.578	2506	13.28	4	1.30	HCP	BCC
Ta	1.430	3290	16.68	5	1.50	BCC	BCC
W	1.367	3695	19.41	6	2.36	BCC	BCC
Re	1.375	3458	21.02	7	1.90	HCP	HCP

Another category is criteria established based on free energy.

Troparevsky et al. [38] used high-throughput calculations of the formation enthalpy ${∆H}_{f}$ of binary compounds to predict specific combinations of elements most likely to form single-phase HEAs:

(11) $$\begin{array}{c} {-T}_{ann}{∆S}_{mix}<{∆H}_{f}<37\;\mathrm{meV}\cdot {atom}^{-1} \end{array}$$

The minimum value of this range is set as the ideal mixing entropy of the alloy system, where $T_{\mathrm{ann}}$ is the annealing temperature set in the experiment, and the maximum value of the formation enthalpy is set as the maximum formation enthalpy at which the alloy does not undergo phase separation due to immiscibility of any element pair. The upper limit of this enthalpy change range (37 meV) is considered to cover all known single-phase alloys and is consistent with the thermodynamic model proposed by Poletti and Battezzati [27].

Senkov et al. [39] established a simple thermodynamic criterion to judge whether intermetallic phases exist in HEAs at equilibrium at a given temperature by comparing the Gibbs free energy of solid solution phases and intermetallic compounds:

(12) $$\begin{array}{c} \kappa_{1}^{cr}(T)>\frac{{\Delta H}_{IMx}}{{∆H}_{MIX}};\;\kappa_{1}^{cr}(T)=\frac{T{\Delta S}_{mix}}{\left|H_{mix}\right|}(1-\kappa_{2})+1 \end{array}$$

where $\kappa_{2}$ is a constant and ${0\leq \kappa }_{2}<1$, defined by the formula ${\Delta S}_{IM}=\kappa_{2}\cdot {\Delta S}_{mix}$.

King et al. [40] also started from the Gibbs free energy of forming intermetallic compounds and single solid solution phases and established a new criterion:

(13) $$\begin{array}{c} \Phi =\frac{{\Delta G}_{SS}}{-\left|{\Delta G}_{max}\right|}\geq 1 \end{array}$$

where ${\Delta G}_{SS}$ is the change in Gibbs free energy when a completely disordered solid solution is formed from a mixture of individual elements; ${\Delta G}_{max}$ is the lowest (intermetallic) or highest (separation) possible Gibbs free energy formed by a binary system composed of mixtures.

This type of criterion predicts phase outcomes by simulating the competition between several metallographic formation stages inside the actual alloy. In this process, the driving force for solid solution phase formation can be easily determined, but it is quite difficult to calculate the driving force for intermetallic compound formation only based on composition. Therefore, in these criteria, the composition of intermetallic compounds is usually assumed to infer their formation driving force.

Due to the differences in alloy types, preparation processes, and heat treatment methods, it is inevitable that there are large discrepancies between theoretical criteria and actual experimental results. In addition, the effectiveness of the criteria themselves also needs to be considered when conducting alloy design. For example, Ghosh et al. [41] found, by comparing the calculation results of the criteria with experimental data, that Formula (5) is too loose in judging whether an alloy can form a solid solution, while the criteria established based on free energy are often too strict in this regard. Moreover, further calculations and designs using density functional theory and thermodynamic principles by Rao et al. [42] have proven the importance of the VEC condition in the design and development of HEAs. Therefore, combining multiple criteria to jointly guide alloy design can often form a reliable theoretical basis.

### 2.2. Simulation Calculation

The broad composition design space of HEAs and the complex interactions between various alloys not only endow HEAs with huge development potential but also increase the time and economic cost of experimental trial and error. Computational materials science-driven design technologies, including molecular dynamics (MD), density functional theory (DFT), calculation of PHAse diagrams (CALPHAD), phase-field modeling (PFM), machine learning (ML), and artificial intelligence (AI), can effectively simulate the phase composition, mechanical properties, electronic structure, and phase transition process of materials, thereby realizing efficient screening of HEA composition design, greatly improving the efficiency of alloy development, and providing strong support for the research of this type of material.

#### 2.2.1. Fe-V-Based HEA Design Based on Density Functional Theory

Density Functional Theory is a computational method based on quantum mechanics to study the electronic structure and properties of many-electron systems. It can accurately calculate electronic structure, bonding characteristics, and alloy energy using ab initio methods, thereby explaining phase stability, structural evolution, and interfacial behavior in HEAs at the atomic scale, which plays an important role in the research of alloy design and performance modulation.

Simulations based on DFT have been able to effectively predict the elastic properties of HEAs. Li et al. [43] used the virtual crystal approximation (VCA) method based on density functional theory results to study the effects of different V concentrations on the structural and elastic properties of Al_0.4_Co_0.5_V_x_FeNi. The calculation results showed that all Al_0.4_Co_0.5_V_x_FeNi-based HEAs have FCC + BCC structures, among which the mechanical stability of the FCC phase is significantly better than that of the BCC phase. When the content of the V element increases from 0.2 to 0.8, the content of the BCC phase in the alloy gradually increases, and the bulk modulus and shear modulus of the alloy gradually decrease. When the content of the V element is 0.8, the Poisson’s ratio of the BCC structure increases abnormally, further indicating that with the increase of V element content, the plastic deformation ability of the material decreases and brittleness increases. Similarly, Hobhaydar et al. [44] established a crystal model of VFeCrW_x_ using density functional theory simulation and the special quasi-random structure (SQS) method, and discussed the effects of W doping amount on the mechanical and electronic properties of the alloy. The results showed that the alloy doped with 8 at% W has the best mechanical properties and ductility. In addition, their team put forward a further explanation for the mechanism of the change in alloy properties based on the characteristics of the electronic structure of the alloy. According to the research results of Liu et al. [45], the increase in covalency of the system leads to an increase in the hardness and strength of the material, while resulting in a decrease in ductility. Based on this, Hobhaydar et al. proposed that seeking a good balance between metallic and covalent bonds by regulating the W doping amount in VFeCrW_x_ alloy can obtain elastic materials with both ductility and strength.

In-depth research on the strengthening mechanism and microstructure of HEA materials based on DFT is also one of the main applications of simulation analysis at present. Koval et al. [46] used density functional theory combined with an evolutionary algorithm for crystal structure prediction to study the elastic and electronic properties of multi-principal element alloys CoCrFeNiX (X = Ti, V, Mn, MnV, Cu, Zr, Nb, Mo, Al, Al_2_). Their research found that the spatial distribution of electron density can explain the anisotropy of elastic modulus exhibited by certain alloys. The general trend is: for alloys with partially filled 3d and 4d elements, the calculated predictions show that the bulk modulus, shear modulus, and Young’s modulus increase with the increase of d-shell occupancy until the half-filled state, with the only exception being a decreasing value of the bulk modulus observed for 3d group CoCrFeNiX alloys. Wu et al. [47] used ab initio methods to study the variation law of stacking fault energy of VNiCo, FeNiCo, and VNiFeCo alloys with long-range order. Their research results showed that with the increase of order, the stacking fault energy increases by 22.2~85.3 mJ/m^2^; the increase in stacking fault energy of paramagnetic V-Ni-Fe-Co alloys is consistent with the local magnetism caused by the decrease of lattice parameters. In addition, modeling HEAs as pseudo-binary systems [48], combined with thermodynamic models to calculate the effects of precipitation phases in alloys, has been proven feasible [49,50], which is of great significance for in-depth understanding of the interaction mechanism between important second phases (such as L12 nanoparticles and Laves phase precipitates) and the matrix in Fe-V-based HEAs. It will bring a more efficient and convenient method for improving the alloy structure and adjusting the strength-plasticity balance of HEAs.

However, the high computational cost of DFT greatly limits its use in alloy development, and the small supercells used in existing studies also make it difficult to conduct research on particle diffusion, lattice stability, and atomic localization. Utilizing the combination of DFT with CALPHAD, molecular dynamics, machine learning, and manufacturing process simulation is crucial for linking atomic-scale mechanisms with macro-scale material behavior.

#### 2.2.2. Fe-V-Based HEA Design Based on Phase Diagram Calculation

The composition, structure, and evolution of phases in alloys have a decisive influence on the various properties of alloys. Therefore, phase design is an indispensable part of HEA development. The CALPHAD method is a computational phase diagram technology based on thermodynamic principles. It predicts phase equilibrium behavior by constructing thermodynamic models of each phase in the material system, and has unique advantages in analyzing the phase stability and phase transition behavior of complex multi-component systems [51].

Simulating and calculating the equilibrium phase diagram of the alloy system to guide phase design [52,53] and prediction [54] of alloys is the most common application of the CALPHAD method. This method can not only help develop HEAs with a single solid solution phase but also significantly enhance the precipitation strengthening effect of HEAs and improve the mechanical properties of materials by designing nano-scale precipitated phases [54,55,56]. In addition, in-depth understanding of the thermodynamic and mechanical properties of alloys through simulation prediction can help improve the stability of high-temperature phases of alloys [57], generate key thermodynamic performance data of alloys [58] and optimize alloy processing methods.

The CALPHAD method has also been widely used in designing the composition of eutectic HEAs and simulating eutectic microstructures [59,60]. Single-phase HEAs often have obvious shortcomings in mechanical properties. Designing eutectic HEAs by regulating the element selection and concentration of alloys to make different crystal phases in the alloy complement each other in performance is an important direction for HEA development. However, HEAs often have extremely complex microstructures [61,62] and multiple crystal phases near the eutectic composition [55,63]. Accurately simulating the microstructure and crystal phase composition of pre-designed eutectic HEAs through the CALPHAD method can effectively improve the design efficiency of alloys and regulate the performance of alloys. Rahul et al. [64] designed Fe_35−x_Co_10_Ni_25_Cr_15_V_10_Mn_x_ eutectic HEAs with FCC and LAVES dual phases. Their team drew a pseudo-binary phase diagram related to the change in Nb concentration through the CALPHAD method, analyzed the phase composition of the eutectic composition domain of this series of alloys, and finally decided to intentionally introduce a softer but more ductile FCC phase and a hard and brittle LAVES phase to achieve complementarity in mechanical properties of the alloy. Characterization results of samples obtained by arc melting showed that the alloy is composed of lamellar two-phase structures. Small changes in Nb content not only have a great impact on the microstructure, such as phase morphology and lamellar spacing but also significantly change the mechanical properties of the alloy.

In the development of Fe-V-based HEAs, the CALPHAD method has been widely used. Saikumaran et al. [65] used the CALPHAD method combined with the Scheil cooling model to predict the microsegregation phenomenon of CrFeMoV alloy during solidification and successfully explained the reason for the formation of dendritic phases in the alloy. Carruthers et al. [66] successfully predicted the phase composition of annealed TiVCrMnFe and Si_0.1_TiVCr_0.5_Fe HEAs and the content of elements in each phase through the calculation of equilibrium phase diagrams, and explained the phase transition mechanism based on the phase diagram results. Choi et al. [67] aimed to seek a more accurate thermodynamic description of the Cr-Fe-Ni-V HEA system and the σ phase in this system. They established a semi-empirical critical evaluation criterion through the CALPHAD method. Ghosh et al. [41] predicted the mixing enthalpy ${∆H}_{mix}$ of HEAs based on the binary Regular Solution Model (RSM) through density functional theory combined with 21 sets of binary special quasi-random structure data. On this basis, 8 empirical models for HEA design were evaluated using the phase structure data of 36 experimentally synthesized low-activation HEAs. The model with better prediction results (Formula (10)) was selected for high-throughput screening. Using this model, 12 equiatomic HEAs that can form simple solid solutions, such as VFeMnW, VCrMnW, VCrTiW were screened out, and the phase diagrams of six of these quaternary alloys were calculated by the CALPHAD method, and the results can well reflect the theoretical prediction. In addition, their team also took the V-Fe-Cr-Ti series alloys as an example to calculate the tendency of the alloy to form a single solid solution under different concentrations of each element. The results showed that the single-phase formation domain in this series of alloys strongly depends on Ti concentration, and when the molar fraction of Ti exceeds 30%, the VFeCrTi alloy cannot form a single solid solution.

The CALPHAD method has strong predictive ability for equilibrium phase diagrams, but it is relatively dependent on the database used for calculation. For multi-component alloys, especially HEAs with more than five components, different databases often have large differences in prediction results. In addition, this method is difficult to effectively predict kinetic transition processes and metastable phases. To address such problems, seeking the combination of the CALPHAD method with other simulation calculation methods will become an effective way for its wider application.

#### 2.2.3. Fe-V-Based HEA Design Based on Molecular Dynamics

The MD method is a widely used computational technique to simulate the time-dependent behavior of atomic and molecular systems. It uses classical mechanics to determine the trajectories of atoms and molecules by solving Newton’s equations of motion. The MD method has gained great attention in HEA design. It provides insights at the atomic scale into alloy behavior under various conditions by studying the physical motion and interaction of particles over time [68]. This method can simulate complex interactions between grain boundaries, dislocations, precipitates, and other structures in materials, providing a powerful means for in-depth understanding of the mechanical properties, phase transitions, and defect dynamics of HEAs [69].

One of the main applications of the MD method in HEA design is to analyze dislocation multiplication, motion, thermodynamic stability, and the interaction between dislocations and other structures in materials. Combining the MD method with nanoindentation technology can simulate the changes in material microstructure over time, analyze the dislocation motion and evolution mechanism at the microscale [70,71], which is of great significance for understanding the work hardening mechanism of materials, studying elastoplastic deformation behavior and its plastic deformation mechanism at the atomic scale [72], and balancing the strength-toughness design of HEAs. Precipitate-strengthened HEAs often have excellent mechanical properties. Simulating the nucleation and growth of precipitated phases, studying the thermal stability of precipitated phases [73], and calculating the diffusion coefficient between dislocations and precipitated phases [74] through the MD method are of great significance for understanding the precipitation strengthening mechanism and further developing this type of alloy.

MD simulation of tensile tests provides an important means for understanding material deformation mechanisms, studying plastic strain evolution, and phase structure transformation. The MD method can effectively simulate the deformation process, grain boundary behavior [75], phase transition [76,77], and dislocation reactions caused by stacking faults [78] of materials under different compositions, phase structures, load conditions, and test temperatures. Chen et al. [79] adopted a research framework combining machine learning and molecular dynamics to study the effect of V addition on the yield strength of Cr-Fe-Co-Ni-based HEAs. To obtain an excellent combination of strength and toughness, their team used MD simulation to obtain the most stable V and Cr content range of the FCC structure in V-Cr-Fe-Co-Ni-based HEAs. In addition, they performed MD simulations of tensile deformation on single-crystal and polycrystalline simulation cells, provided data sets for 9 different ML models respectively and verified the results, and compared them with previous experimental studies to verify an optimal model. Based on this model, they calculated that V5Cr16Fe9Co35Ni35 has the most excellent mechanical performance, and the events observed during MD simulation of plastic deformation are quite consistent with previous experimental results. In addition, existing simulation results show that the deformation mechanism and mechanical behavior of HEAs are highly sensitive to interfacial plasticity. For example, Ma and Liu et al. [80,81] found that interfacial coherence can promote dislocation slip, while semi-coherence often leads to dislocation accumulation and amorphization [82]. Therefore, MD simulations focusing on interfacial coherence simulation provide a new method for the design optimization of HEAs. In addition to conventional tensile tests, MD methods have also achieved extensive and effective simulations of creep properties of nanocrystalline HEAs [83], microstructure evolution of HEAs under impact load [84], tribological behavior of HEA coatings [85], phase transition of metastable HEAs [86], crack propagation, and lattice disorder of HEAs under cyclic deformation [87].

Studying the defect evolution, phase transition process, and phase stability of HEAs during smelting, processing, and heat treatment through the MD method is also of great significance for the mechanical property design and industrial preparation of HEAs. Simulating the process from the molten state to the crystalline phase in HEAs through the MD method can deeply explore the effects of factors such as the content of different elements and cooling rate on diffusion transformation during material solidification, as well as grain nucleation and growth during phase transition [88,89]. In addition, the MD method has been widely studied in simulating the rapid solidification process of high-entropy alloys (HEAs) at the atomic scale. These rapid solidification simulations systematically study the element distribution, temperature field change, atomic diffusion, displacement trajectory, and structural evolution during solidification [90]. In addition, some material processing methods, such as metal coating preparation [91] and laser shock peening [92], are often difficult to perform performance testing due to the particularity of their processing principles, while the MD method provides an effective simulation for these processing processes. Its simulation can not only provide a means for mechanical performance testing, composition, and structural analysis of materials but also is of great significance for the research on the generation, migration, and evolution mechanisms of internal defects in materials.

The MD method has also achieved extensive academic results in simulating the irradiation damage behavior of HEAs, providing a simpler, more efficient, and economical research method for the design of irradiation resistance of this type of material. The MD method can simulate the material irradiation damage behavior caused by various types of irradiation particles such as electrons, helium ions, heavy ions, and neutrons, and can obtain different irradiation conditions by adjusting particle energy, irradiation temperature, and simultaneous irradiation of multiple types of particles. Through simulation, the generation and migration of material defects during irradiation [93], evolution of dislocation loops [94], diffusion behavior related to composition segregation [95], phase structure stability, crack healing behavior, and anisotropic mechanical behavior of stacking fault tetrahedrons [96]. can be clearly understood. In addition, significant achievements have been made in the simulation of primary damage in HEAs. Experimental results by Deluigi et al. [97] show that the origin of radiation resistance of HEAs may not be related to the reduction of primary damage caused by chemical disorder, but may be caused by long-term defect evolution. The simulation of irradiation damage behavior by the MD method provides a new solution for many experiments that cannot be carried out due to sample limitations, and is also of great significance for understanding the relationship between lattice distortion and sluggish diffusion effect of HEAs and irradiation resistance. This simulation method has great application potential in the design of irradiation resistance of HEAs.

The MD method allows us to simulate larger system sizes and longer time scales. The key to its effective implementation is to use reliable empirical interatomic potentials to calculate system energy and interparticle interaction forces. The quality of MD simulation mainly depends on the accuracy of empirical potentials. At present, MD simulation of HEAs is relatively dependent on existing interatomic potentials, and the interatomic potentials of HEAs as multi-component alloys are often more complex, so their effectiveness also needs to be considered.

#### 2.2.4. Fe-V-Based High-Entropy Alloy Design via Phase-Field Modeling

Phase-field modeling (PFM) is a mesoscale numerical calculation model based on continuum mass density functional theory. It simulates the evolution of microstructures in high-entropy alloys by tracking the spatiotemporal changes of phase boundaries. This method can effectively simulate and predict the spatial and temporal evolution of microstructure morphological patterns, as well as their response to multi-physical fields and associated properties.

PFM provides key insights into the complex phase transformations and microstructural evolution of HEAs, helping to optimize alloy compositions and processing parameters to achieve desired properties. It has been successfully applied to predict the composition [98] and microstructural evolution [99,100] of HEAs. Kamalnath et al. [101] investigated the effects of two spinodal decomposition-based phase transformation paths (PTPs) on microstructural evolution in HEAs via phase-field simulation. Their team focused on exploring the influences of equilibrium volume fraction, free energy asymmetry, and elastic modulus mismatch on HEA microstructures, and adopted a combined constitutive thermodynamic tensor model to extract thermodynamic data related to phase coherence and microstructural evolution of multiple components. This research breaks through the understanding of traditional nucleation-growth-based phase formation mechanisms and systematically reveals the regulatory mechanisms of key parameters on HEA microstructures in spinodal decomposition-mediated phase transformation paths. The high-precision simulation results of phase transformations provided by PFM fully demonstrate its predictive capability for complex systems such as HEAs. PFM’s powerful phase evolution simulation capability also makes it important for HEA smelting [102,103,104]. Combining PFM with experimental data and other simulation methods (e.g., molecular dynamics, CALPHAD) enables a more comprehensive understanding of the solidification process of HEAs under non-equilibrium conditions and the densification behavior during sintering.

PFM also plays a crucial role in studying the elastic properties, as well as inelastic behaviors such as yielding and hardening of HEAs. By analyzing changes in the energy landscape caused by compositional fluctuations in materials, we can further investigate the morphological evolution and critical stress during dislocation motion. Lauren et al. [105] utilized a phase-field dislocation dynamics model with atomic-level information to conduct an in-depth study on the effects of the ratio of screw to edge dislocations and lattice energy distribution on the morphological transformation and critical stress of long screw dislocations during slip in refractory MPEAs. Their team showed that changes in lattice energy—whether in content or scale—induce strain hardening-like behavior in the alloy, i.e., the critical stress for activating slip increases with slip distance. Moreover, under different magnitudes of lattice energy change, dislocations move and evolve in two distinct ways.

PFM also exhibits unique advantages in explaining certain irradiation damage behaviors and their corresponding mechanisms. Its efficient simulation of phase evolution enables effective modeling and analysis of irradiation-induced precipitation. Niu et al. [106] adopted a phase-field method based on the Cahn–Hilliard equation and a radiation-enhanced diffusion model to simulate the effects of chemical composition and irradiation conditions on phase separation in nuclear-grade Fe-Cr-Al-based alloys. Ge et al. [107] established a phase-field model for He bubble evolution through a multi-scale computational framework, combining first-principles calculations of defect thermodynamics and helium kinetic results, and systematically analyzed helium behavior in AlNbTiVCr_x_ (x = 0, 0.5, 1) HEAs. Their simulation results indicated that the most effective strategy to control He bubble growth in this series of alloys is to design kinetic pathways for helium atoms. By adjusting the compositional complexity of the alloy to manipulate defect dynamics, microstructural evolution can be controlled, thereby enhancing the irradiation swelling resistance of HEAs.

PFM can effectively obtain mesoscale structural evolution; however, its simulation accuracy depends on input parameters such as interfacial energy and mobility, which are often difficult to obtain for HEAs. This is one of the main reasons for the limited application of phase-field simulation in Fe-V-based HEAs at present. In recent years, with the development of multi-scale computational frameworks, this issue has gradually been resolved. Whether it is the generation of pre-required parameters for PFM simulations via DFT, MD, and CALPHAD, or machine learning (ML) accelerating computational simulation processes, PFM is increasingly becoming an important tool for optimizing the structure and performance design of Fe-V-based HEAs.

#### 2.2.5. Comparison and Integration of the Computational Approaches

**Figure 2:**
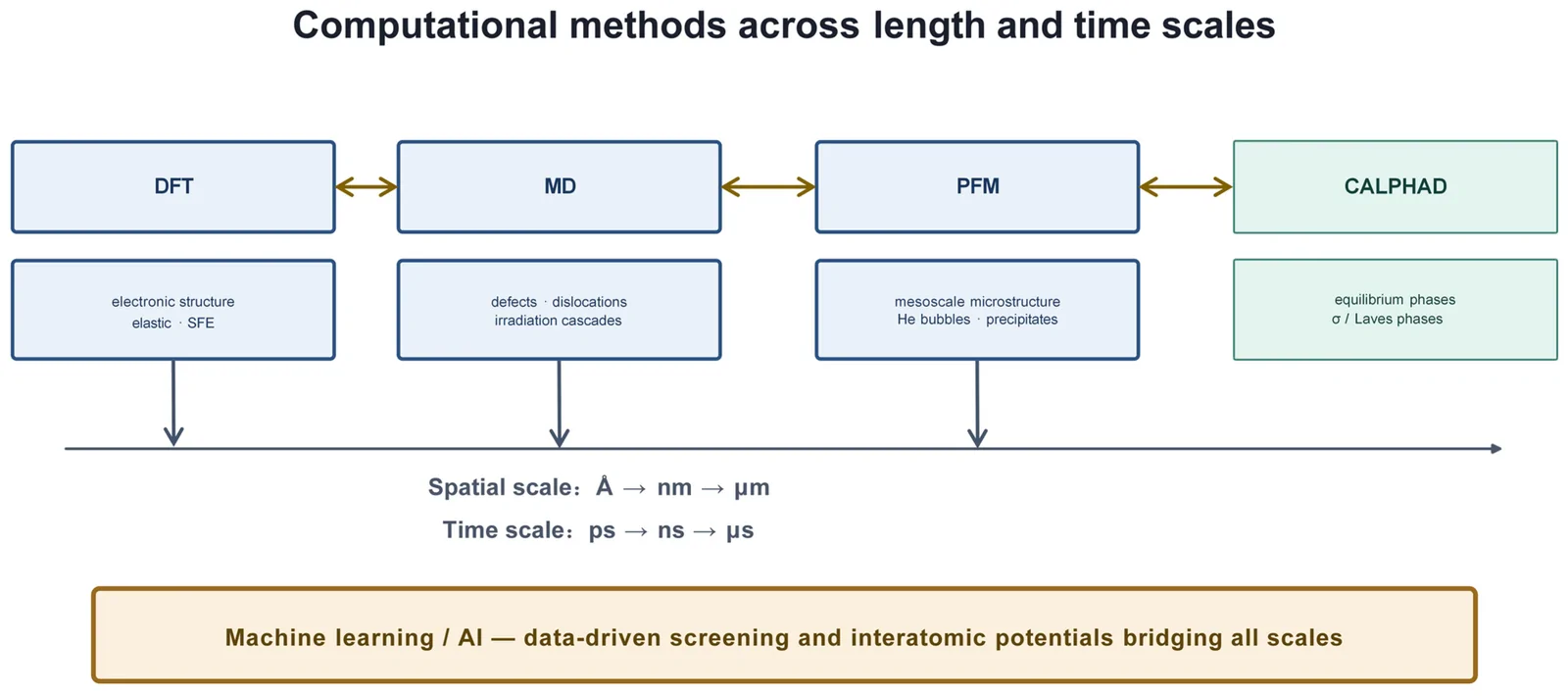
Computational methods across length and time scales. DFT, MD, PFM and CALPHAD cover complementary domains from the electronic to the mesoscale, while machine learning bridges these scales through data-driven screening and interatomic potentials.

The four approaches complement one another across spatial and time scales, but each occupies a distinct niche, so which performs best depends on the target property and scale. For phase stability, elastic constants and stacking-fault energy, DFT is the most reliable ab initio tool and has now been applied to more than 500 alloy property combinations across HEAs and compositionally complex alloys; it is nevertheless limited by finite-temperature excitations (electronic, magnetic and vibrational), by the paramagnetic state of most Fe-V alloys, which non-magnetic DFT cannot capture, and by chemical short-range order [108]. CALPHAD offers fast equilibrium screening but is database-dependent and cannot capture kinetics or metastable phases. Molecular dynamics resolves defect and cascade dynamics but depends on empirical potentials that remain scarce for Fe-V systems. The most pressing remaining challenges are scarce Fe-V potentials and databases, the cost of finite-temperature and magnetic DFT, and fragmented data [109]. Figure 2 shows the applicable ranges of different computational methods.

## 3. Preparation

The composition and proportion of elements determine the inherent properties of the alloy, while the preparation process has a key impact on the microstructure, composition uniformity, and internal defects of the alloy, and also has a decisive effect on the alloy properties. The main preparation methods of Fe-V-based HEAs include vacuum arc melting (VAM), vacuum induction melting (VIM), powder metallurgy, laser cladding (LC), additive manufacturing(AM), and magnetron sputtering.

### 3.1. Direct Melting Preparation Method of HEAs

Vacuum arc melting is one of the most common smelting methods for preparing Fe-V-based HEAs. Under an argon protective atmosphere, the sample is directly melted by a high-temperature arc, and the copper crucible combined with a water circulation system is used to quickly cool the sample. Arc melting has high preparation efficiency, a simple preparation process, and almost no requirements on the shape and particle size of raw materials. Cui et al. [110] prepared FeCrV multi-principal element alloys by arc melting. The as-cast alloy is composed of a single BCC phase. Through analysis of EBSD and IPF results, it can be found that the as-cast FeCrV sample has a large number of columnar crystals, whose direction is perpendicular to the solidification direction.

During vacuum arc melting, the grain structure at different positions of the material is uneven due to different cooling rates, accompanied by a certain degree of micropores. To solve this problem, homogenization annealing is often necessary after arc melting. Sun [111] prepared Fe-Ti-Cr-V-W HEAs with different atomic ratios in as-cast and annealed states, respectively. Among them, non-equiatomic alloys maintain a single BCC solid solution structure before and after annealing. After annealing, the alloy hardness (HV0.2) increased from 5.45 GPa to 6.56 GPa while maintaining toughness unchanged; yield strength (σy) increased from 950 MPa to 1150 MPa, fracture strength (σu) increased from 1309 MPa to 1501 MPa, and the mechanical properties of the alloy were improved. Luo et al. [112] prepared Fe_36.41_Cr_28.06_V_35.53_ medium-entropy alloy. After homogenization treatment, partial segregation of the alloy was dissolved into the matrix, dendrites were reduced, the degree of matrix segregation was weakened, and the mechanical properties of the alloy were improved. Tests showed that the hardness (HV0.2) of the as-cast alloy was 4369.82 MPa, and the hardness of the alloy after homogenization treatment increased to (HV0.2) 4628.54 MPa, and the yield strength (σy) increased from 789.7 MPa to 988.3 MPa.

Vacuum induction melting is also a preparation process for obtaining target alloys by directly melting raw materials. This method uses a high-frequency coil to generate an alternating magnetic field, so that the molten metal is suspended inside the coil without contact with the metal container. Compared with vacuum arc melting, this method can smelt larger-volume samples at one time, easier control of active elements, offers faster melting speed, less smelting loss, and can greatly avoid contamination of the melt by crucible materials [113,114].

### 3.2. Powder Metallurgy Preparation Method of HEA

HEA powder metallurgy includes two steps: mechanical alloying (MA) and sintering. Mechanical alloying is a process in which powder particles are mixed and then continuously diffused between reactive elements under the mechanical action of ball milling media to obtain uniform nanocrystalline powder [115,116]. Common sintering methods include spark plasma sintering (SPS), vacuum hot-pressing sintering (VHPS), and hot isostatic pressing (HIP). SPS technology achieves high densification of powder in a short time by applying a large DC pulse current and high pressure simultaneously [117]. Due to the short sintering time, fast heating speed, and low sintering temperature, nanocrystalline structures are more likely to form in the alloy, thereby endowing the material with excellent mechanical properties. In the VHPS process, the powder is in a thermoplastic state during heating and pressing, which is conducive to the diffusion and mass transfer process of particles, so the forming pressure is greatly reduced compared with cold pressing. This feature not only reduces the sintering and tempering temperature but also shortens the sintering time, effectively inhibits grain growth, and finally obtains a finished product with fine grains and high density. In the HIP process, during sintering, argon or nitrogen is uniformly applied as a pressure medium through a high-pressure air pipe, combining high temperature and high pressure. Unlike VHPS, which applies pressure only in a uniaxial direction, HIP applies uniform force in all directions, thereby obtaining a dense sintered body and eliminating the need for pressure transmission through rigid molds. HIP can effectively reduce the sintering temperature, shorten the sintering time, and inhibit grain growth. During powder alloying, the grains are repeatedly crushed and cold-welded. This preparation method can overcome the composition design limitations of conventional smelting methods, obtain a uniform nanoscale crystal structure, and thus make the alloy have favorable mechanical properties. On this basis, combined with appropriate sintering methods, grain growth can be further inhibited, solidification defects such as delamination and segregation can be avoided, and a uniform fine-grained structure can be obtained.

In the powder metallurgy process, factors such as ball milling time of metal powder and sintering temperature will greatly affect the composition uniformity and micromorphology of the obtained alloy, thereby causing differences in alloy properties. Alijani et al. [118] prepared FeCoNiMnV HEAs by mechanical alloying and spark plasma sintering, and studied the microstructure, phase evolution, and mechanical properties at different sintering temperatures. The results showed that at different sintering temperatures from 500 °C to 1000 °C, with the increase in sintering temperature, the original BCC + FCC phase structure of the alloy tends to transform into a new FCC phase and a tetragonal phase dominated by V and Mn. In addition, the increase of sintering temperature reduces the number of pores in the alloy, improves the density, and refines the grains of the alloy, so that the alloy obtains the smallest grain size and the highest hardness at a sintering temperature of 900 °C. Yurkova et al. [119] prepared two equiatomic nanoscale HEAs, AlCoFeCrVNi and AlCoFeCrVTi, by combining mechanical alloying and hot pressing sintering. With the gradual extension of ball milling mixing time, the XRD analysis results of the powder showed that the phase composition of the powder gradually transformed from multiple pure element phases to a single solid solution phase. After 10 h of ball milling and pressure sintering at 5 GPa, 800 °C for 30 min, both formed an FCC + BCC phase with an average grain size of ~85 nm. After mechanical testing, it was found that both HEAs have excellent mechanical properties (σy > 3000 MPa, ε > 25%).

### 3.3. Laser Cladding Preparation Method of HEAs

Laser cladding (LC) is an important coating preparation process, which has been widely used in fields such as part repair and remanufacturing, surface modification, and mechanical manufacturing. Laser cladding uses a high-energy laser beam to irradiate the metal powder coating on the surface of the cladding substrate. The coating material and the substrate surface melt at high temperature and then solidify rapidly, making the substrate and the surface coating form a good metallurgical bond. The HEA coating prepared by laser cladding presents a non-equilibrium solidification state due to the fast cooling rate, and the different grain morphologies from the substrate to the coating surface make it have the characteristics of hard outside and tough inside, thereby significantly improving the wear resistance, corrosion resistance, and oxidation resistance of the substrate surface [120,121].

Song et al. [122] studied the microstructure evolution of CoCrFeNiNbV_x_ (x = 0, 0.5, 1, 1.5) HEA laser cladding layers and their effects on room-temperature and high-temperature wear resistance by regulating V content. The results showed that all cladding layers exhibited typical hypereutectic morphology, and with the increase of x, the stability of the FCC phase decreased, and the phase composition gradually transitioned from FCC + Laves + NbC to BCC + Laves. The microhardness of the cladding layer was significantly higher than that of the substrate. With the increase of x, the microhardness, room-temperature wear resistance, and high-temperature wear resistance of the cladding layer first increased and then decreased, reaching the best value at x = 1, which were 1.61 times, 7.04 times, and 7.40 times higher than those at x = 0, respectively. Liu et al. [45], starting from ab initio calculations and experimental results, prepared and studied the effects of Si on the crystal structure, electronic structure, mechanical properties, and tribological behavior of the FeCrMnVSi_x_ (x = 0.5, 1.0, 1.5, 2.0) HEA system by laser cladding. The results showed that the addition of Si atoms increased the degree of lattice distortion and the proportion of covalent bonds in the alloy. Calculated mechanical properties and experimental measurement results showed that the introduction of Si improved the elastic properties and hardness of FeCrMnVSi_x_. Among them, the ultimate tensile strength of FeCrMnVSi_2.0_ was 50% higher than that of FeCrMnV, while its elongation and yield strength were almost unchanged. Due to the increase in the hardness of HEAs and the friction reduction effect caused by the formation of SiO_2_ layer during friction, the tribological properties of FeCrMnVSi_x_ were also far superior to the substrate. The friction coefficient and wear weight loss rate of FeCrMnVSi_2.0_ were only 30% and 25% of those of the substrate, respectively.

The LC process can significantly improve surface performance without affecting the properties of the substrate, but due to the limited size of the cladding layer and relatively high cost, this method is not suitable for large-area cladding at present. At the same time, due to the temperature gradient and different thermal expansion coefficients between the cladding layer and the substrate material, as well as unmelted particles and accumulated gas in the cladding layer, the LC process may produce various defects in the cladding layer, such as pores, cracks, deformation, and surface unevenness [123]. Some studies adopt heat treatment to improve the internal defects of the cladding layer. Appropriate heat treatment can not only release a large amount of residual stress in the cladding layer but also produce harder new phases to further improve the hardness and wear resistance of HEAs.

### 3.4. Additive Manufacturing Preparation Method of HEAs

Additive manufacturing technology uses alloy powder as raw material and directly manufactures parts under the action of a high-energy laser beam or electron beam combined with 3D data of parts. Compared with cast HEA parts prepared by traditional processes, additive manufacturing can prepare HEAs with complex morphologies, realize the transformation and repair of damaged parts and tools, and manufacture multi-metal composite workpieces. Ultra-fast solidification under non-equilibrium conditions can refine grain size and form ultra-fine crystalline structures. Common additive manufacturing technologies for HEAs include powder bed fusion (PBF), laser directed energy deposition (DED), and binder jetting printing (BJP) [124]. Laser powder bed fusion (PBF) can be divided into laser powder bed fusion (LPBF) and electron beam powder bed fusion (EPBF). Laser directed energy deposition (DED) is divided into directed energy deposition–laser beam (DED-LB), directed energy deposition–electron beam (DED-EB), and directed energy deposition–arc melting (DED-Arc) [125].

PBF technology manufactures HEAs by melting raw material powder in selected areas of the metal powder bed layer by layer. This technology can process parts with complex shapes and directly form the final part from a 3D model without subsequent processing [126]. Yao et al. [127] prepared almost fully dense Al_0.5_Cr_0.9_FeNi_2.5_V_0.2_ HEAs with an FCC matrix and L1_2_ nanophases by the SLM method. Their research results showed that the high cooling rate and non-equilibrium solidification of the SLM method lead to a layered heterogeneous structure composed of columnar grains, subgrains, L1_2_ nanophases, and dislocations inside the alloy, which helps to improve strength without losing ductility. Subgrains significantly improve the strength of the alloy through dislocation hardening, while heterogeneous structures at different levels hinder dislocation movement and enable gradual deformation of the material, thus achieving a balance between strength and plasticity of the alloy.

DED technology transports metal powder synchronously with high-energy beams to form a molten pool on the substrate and deposit materials layer by layer. This method is mostly used for surface strengthening of parts, morphology modification, repair of damaged parts, and processing and manufacturing of multi-material composite workpieces [128,129,130]. Gwalani [131] manufactured compositionally graded HEAs AlCrFeMoV_x_ (x = 0.0~1.0) by DED technology. The results showed that the graded alloy exhibited a single BCC structure; V had good solubility in the alloy and could effectively improve the hardness of the alloy (19.8%), and subsequent annealing treatment results showed that the material had good phase stability. HEAs prepared by DED technology have high forming accuracy and good surface quality but low forming efficiency and limited forming size.

Binder Jetting Printing (BJP) technology bonds powder materials layer by layer by jetting binder through inkjet print heads, and then obtains the final parts through degreasing and sintering. This technology has the advantages of high production efficiency, a simple process, and wide material application, but has high requirements for the selection of binders. At present, only a small number of materials can effectively apply this technology [132,133], and the material shrinkage caused by degreasing during manufacturing is also difficult to control and predict.

In the additive manufacturing of Fe-V-based HEAs, the rapid cooling process not only results in finer grains but also easily leads to thermal stress concentration, thereby increasing the probability of crack generation in the material. Improper setting of high-energy beam scanning parameters can also easily cause pores or unmelted areas inside the material, thereby affecting density and mechanical properties. Effective scanning strategies can greatly reduce manufacturing defects of Fe-V-based HEAs prepared by additive manufacturing and improve the internal microstructure of materials [134]. In addition, for Fe-V-based HEAs prepared by additive manufacturing, most studies focus on mechanical properties such as hardness. However, research on their fatigue life, fracture toughness, and corrosion resistance has not been fully explored.

### 3.5. Magnetron Sputtering Preparation Method of HEAs

Magnetron sputtering refers to a process in which, under vacuum or a specific atmosphere, plasma is used to bombard the target material under the influence of a magnetic field, and the sputtered target atoms are deposited on the substrate to form a film. It is often used for surface modification of precision parts and can significantly improve the wear resistance, corrosion resistance, or high-temperature resistance of parts [135,136]. Magnetron sputtering has unique advantages in the preparation of HEA ceramic films and amorphous structure formation.

Oxidation is prone to occur during the preparation of Fe-V-based HEA films, so there are high requirements for the ambient atmosphere of magnetron sputtering. Taking this as the research object, Liu [137] and Zhang [138] et al. successively studied the effects of N_2_ and O_2_ in the DC magnetron sputtering ambient atmosphere on the film morphology, chemical composition, microstructure, and mechanical properties of AlFeCoNiCuZrV HEA films. The results showed that the films exhibited an amorphous structure under any ambient atmosphere. Properly increasing the proportion of a certain gas in the ambient atmosphere would make the film surface smoother, while the thickness of the film would gradually decrease with the increase in the proportion. To explain the above phenomena, their team pointed out that, on the one hand, the reaction between gas molecules and alloy elements leads to the formation of metal compounds in the film, the internal stress of the film increases, and the deposition efficiency decreases; on the other hand, the reaction between gas molecules and the target material leads to the deposition of compounds on the target surface, hindering the deposition of particles, i.e., target poisoning. In addition, different proportions of the two gases in the ambient atmosphere have different effects on the mechanical properties of the film. Overall, adding an appropriate amount of N_2_ to the atmosphere has a positive effect on the Young’s modulus and hardness of the alloy film, while the addition of O_2_ will seriously damage the mechanical properties of the film.

Magnetron sputtering can often prepare amorphous HEA films, while Dou et al. [139] prepared FeAlCoCuNiV HEA coatings with a single FCC structure by DC magnetron sputtering, whose hardness and Young’s modulus can reach 19.7 GPa and 18.9 GPa, respectively, and the corrosion resistance is better than that of 201 stainless steel. HEA coatings have good application prospects as anti-corrosion protective coatings to replace stainless steel. In the initial stage of film preparation by magnetron sputtering, the structure of the film is controlled by the substrate, making the microstructure of the film prone to non-crystallization. When the thickness of the film increases to a certain extent, the influence of the substrate on the film becomes smaller, so the film transforms into its inherent solid solution structure. From the research results of Kalita et al. [140], it can be seen that due to the influence of the grain orientation of the substrate material, the film formed by magnetron sputtering shows two morphologies: flat and serrated under SEM observation. With the continuation of the deposition process, the initially uniform region gradually breaks into nanopillars mainly separated by low-angle grain boundaries (LAGBs), and gradually develops into a single BCC solid solution phase.

HEA coatings prepared by magnetron sputtering have the advantages of high density, high deposition efficiency, and strong performance stability, but also have problems such as low target utilization rate, high requirements for target purity, limited coating thickness, and low adhesion between the coating and the substrate. Aiming at the existing problems, certain solutions have been developed in the industry: adding a transition layer between the substrate and the coating can alleviate the problem of low film adhesion; increasing the substrate temperature to enhance the diffusion ability of atoms to be sputtered into the substrate, thereby improving adhesion; performing heat treatment on the sputtered sample to strengthen atomic diffusion between the coating and the substrate and improve adhesion. In addition, the bias voltage of the process parameters of magnetron sputtering affects the microstructure of the HEA coating and thus affects the performance of the coating, and the coating thickness also has a certain influence on the mechanical properties of the coating.

## 4. Room-Temperature Mechanical Properties

As one of the candidate materials for reactor structural materials, a large number of studies on Fe-V-based HEAs focus on their mechanical properties. For existing Fe-V-based HEAs, the fracture strain in tensile tests is usually much lower than that in compression tests [141], which is related to the tensile brittleness caused by the lattice distortion effect of HEAs themselves and slow kinetics [10,142]. Table 2 is a list of comprehensive mechanical properties of Fe-V-based HEAs at room temperature, including the preparation method, phase composition, yield stress, plasticity, and microhardness of various materials.

**Table 2:** Summary of structures and mechanical properties of Fe-V-based HEAs at room temperature.

Materials	Phase Composition	Smelting Method ^#^	Yield Stress (MPa)	Elongation (%)	Hardness (HV)	Ref.
FeCrV	BCC	MA	1850	28	555	[143]
FeCrVCu_0.05_	BCC	MA	2000	35	630	[144]
FeCrVTi_0.2_	BCC	MA	1910	35	595	
CrFeVCu	BCC	VAM-AS	727	53.8	383	[145]
CrMn_0.3_FeVCu_0.06_	BCC + FCC	VAM-AS	1273	50.7	451	
CrMn_0.5_FeVCu_0.1_	BCC + FCC	VAM-AS	1087	49.2	473	
CrMn_0.7_FeVCu_0.14_	BCC + FCC	VAM-AS	1068	49.8	445	
CrMnFeVCu_0.2_	BCC + FCC	VAM-AS	862	48.7	424	
FeCrCoNi	FCC	VAM-HO	190	No fractured	134	[146]
CoCrFeNiV	σ + FCC	VAM-HO	1435	2.5	587	
FeCrCoNiMnV	σ + FCC	VAM-HO	1660	0.5	636	
FeCrV	BCC	VAM-HO	1149	37.6	4.65 *	[110]
FeCrVTi_0.05_	BCC + LAVES	VAM-HO	1493	47.73	4.96 *	
FeCrVTi_0.1_	BCC + LAVES	VAM-HO	1415	27.05	5.39 *	
FeCrVTi_0.2_	BCC + LAVES	VAM-HO	1407	30.43	6.28 *	
FeCrVTi_0.3_	BCC + LAVES	VAM-HO	1393	29.94	6.66 *	
FeCr_2_V	BCC1 + BCC2	VAM-AS	1107	12.5	445	[147]
FeCr_2_VW_0.1_	BCC1 + BCC2	VAM-AS	1189	15	603	
FeCr_2_VW_0.3_	BCC1 + BCC2	VAM-AS	1452	30	714	
Fe_65_Cr_15_Mn_20_	BCC + σ	VAM-AS	290	>79.5	214.0	[19]
V_10_Fe_55_Cr_15_Mn_20_	BCC + σ	VAM-AS	426	>71.3	224.9	
V_20_Fe_45_Cr_15_Mn_20_	BCC + σ	VAM-AS	648	2.3	286.6	
V_30_Fe_35_Cr_15_Mn_20_	BCC + σ	VAM-AS	748	>75.1	380.6	
V_40_Fe_35_Cr_15_Mn_20_	BCC	VAM-AS	992	38.0	463.9	
V_50_Fe_35_Cr_15_Mn_20_	BCC	VAM-AS	1189	25.2	528.4	
V_60_Fe_35_Cr_15_Mn_20_	BCC	VAM-AS	666	31.0	319.6	
FeCrMoV	BCC1	VAM-AS	900	28.2	720	[148]
FeCrMoVTi_0.5_	BCC1 + BCC2	VAM-AS	1520	5.9	818	
FeCrMoVTi_1.0_	BCC1 + BCC2	VAM-AS	1295	5.3	887	
FeCrMoVTi_1.5_	BCC1 + BCC2	VAM-AS	1105	4.7	735	
FeCrMoVTi_2.0_	BCC1 + BCC2	VAM-AS	880	4.2	685	
AlCoFeCrVNi	FCC + BCC	MA	3450	28.6	12.6 *	[119]
AlCoFeCrVTi	FCC + BCC	MA	4100	24.3	14.7 *	
Fe_2_CoNiV_1.0_	FCC	VAM-AS	211.3	60.0	203.4	[149]
Fe_2_CoNiV_1.1_	FCC	VAM-AS	249.3	43.5	206.1	
Fe_2_CoNiV_1.2_	FCC + σ + κ	VAM-AS	274	32.9	224.1	
Fe_2_CoNiV_1.3_	FCC + σ + κ	VAM-AS	315.5	34.3	288.5	
Fe_2_CoNiV_1.4_	FCC + σ + κ	VAM-AS	460.1	8.0	337.6	
FeCoNiV	FCC	VAM-HO	284	30	-	[150]
(FeCoNiV)_94_Al_6_	FCC + BCC	VAM-HO	420	25	-	
(FeCoNiV)_89_Al_11_	FCC + BCC	VAM-HO	810	7	-	
(FeCoNiV)_84_Al_16_	BCC	VAM-HO	1000	2	-	
(FeCoNiV)_80_Al_20_	BCC	VAM-HO	700	5	-	
AlCoCrFeNi	BCC	VAM-AS	1378.9	22.7	534	[151]
AlCoCrFeNiV_0.2_	BCC	VAM-AS	1492.4	26.8	557.6	
AlCoCrFeNiV_0.5_	BCC	VAM-AS	1597.7	5.1	591.3	
AlCoCrFeNiV_0.8_	BCC	VAM-AS	1695.8	5.7	624.8	
AlCoCrFeNiV_1.0_	BCC	VAM-AS	1726.5	0.7	648.8	
FeCrV	BCC	VAM-HO	587	No fractured	645	[152]
Fe_34.9_Cr_29.6_V_35.5_	BCC	VAM-HO	859	No fractured	518	
CrCuFeTiV	BCC + FCC + LAVES	VAM-HO	1510	2.3	618	[153]
VCrFeTa_0.1_W_0.1_	BCC	VAM-HO	1341	42.2	564	[154]
VCrFeTa_0.2_W_0.2_	BCC	VAM-HO	1742	35.7	673	
VCrFeTa_0.4_W_0.4_	BCC1 + BCC2 + LAVES	VAM-HO	1580	2.4	886	
Cu_5_Cr_35_Fe_35_V_20_Ti_5_	BCC + LAVES	VAM-HO	1290	43	590	[155]
Cu_5_Cr_35_Fe_35_V_20_Ta_5_	BCC + Fe_7_Ta_3_	VAM-HO	1000	No fractured	560	
Cu_5_Cr_35_Fe_35_V_20_W_5_	BCC	VAM-HO	800	No fractured	470	
CrMn_0.3_FeVCu_0.06_	BCC + FCC	MA	1405	36.8	5.27 *	[156]
CoFeNiV	FCC	VAM-AS	428.3	No fractured	238	[157]
CoFeNiVMo_0.2_	FCC + CoMo_2_Ni	VAM-AS	686.9	No fractured	267	
CoFeNiVMo_0.4_	FCC + CoMo_2_Ni	VAM-AS	1466.7	19.7	402	
CoFeNi_2_VMo	FCC + Co_2_Mo_3_	VAM-AS	679.1	24.7	382	
FeCrMnV	BCC	SLM	187	20	239	[45]
FeCrMnVSi_0.5_	BCC	SLM	211	18	328	
FeCrMnVSi_1.0_	BCC	SLM	199	16	392	
FeCrMnVSi_1.5_	BCC	SLM	262	17	491	
FeCrMnVSi_2.0_	BCC	SLM	216	19	649	
V_35_Ti_35_Fe_15_Cr_10_Zr_5_	BCC1 + BCC2	VAM-HO	918	0.67	554	[158]

* The hardness data of this material was obtained through nanoindentation tests, with the unit being GPa. ^#^ VAM-AS indicates the as-cast alloy; VAM-HO indicates that the alloy has undergone homogenization annealing treatment.

The strength and ductility of HEAs are influenced by multiple factors, among which the type and content of alloying elements have a decisive impact on the room-temperature mechanical properties of Fe-V-based HEAs. The composition of HEAs determines their plastic behavior and interatomic interactions, which in turn determine their dislocation behavior. In addition, composition also has a decisive effect on alloy phase formation and phase fraction, and subsequently influences properties through microstructure [159]. Aluminum (Al) is one of the most common alloying elements in existing HEA systems. With a valence electron concentration (VEC) of 3, according to Formula (4) and its criteria, the presence of Al in Fe-V-based HEAs often promotes the transformation of the material’s phase composition to the BCC phase, thereby increasing alloy strength while impairing toughness—this is intuitively reflected in the research of Ye et al. [150]. In common Fe-V-based HEA systems, Nb and Ti typically have larger atomic radii compared to other components, thus providing strong solid solution strengthening effects to significantly improve alloy strength [110,148]. However, it should be noted that Ti exhibits an HCP structure at room temperature; excessive addition of Ti in alloys not only weakens the strengthening effect but also tends to form intermetallic compounds, leading to alloy embrittlement [160]. Due to its positive mixing enthalpy and weak bonding with most other elements in Fe-V-based HEAs, Cu often forms Cu-rich segregated phases in interdendritic regions. Cu tends to promote liquid phase separation before solidification and facilitates the formation of Cu-rich supersaturated solid solutions with nanoscale precipitates [161]; the Cu-rich phase weakens the strength of HEAs compared to other Cu-lean phases. In general, adding substitute elements to the original Fe-V-based HEA system can improve yield strength to a certain extent, but the element addition amount must be strictly controlled to avoid performance loss caused by alloying. Furthermore, adjusting the type and content of alloying elements to modify mechanical properties often fails to simultaneously balance yield strength and plasticity.

The preparation method also has an extremely important impact on the mechanical properties of materials. It not only determines the microstructure of the alloy but also introduces specific defects due to the limitations of the processing method itself, thereby greatly affecting the strength and toughness of HEAs. Although direct melting methods such as vacuum arc melting and vacuum induction melting are commonly used, as-cast samples often have relatively coarse dendrites due to the limitations of the melting process. Samples prepared by powder metallurgy methods contain nanocrystals and high-density dislocations internally, leading to a significant improvement in alloy mechanical properties. According to the research of Yurkova et al. [119], the yield strength and toughness of AlCoFeCrVNi and AlCoFeCrVTi prepared by powder metallurgy reached 3450 MPa, 4100 MPa and 28.6%, 24.3%, respectively. Both materials exhibit excellent toughness while possessing impressive yield strength. In addition, even for the same preparation process, adjusting control parameters such as cooling rate and adopting different thermomechanical processing methods (e.g., homogenization, forging, cold rolling, hot rolling) can also influence alloy mechanical properties. Luo et al. [112] prepared Fe_36.41_Cr_28.06_V_35.53_ alloy with a BCC phase via arc melting and sequentially subjected the samples to homogenization annealing, hot rolling, and post-rolling heat treatment. Comparing the as-cast sample with three post-processed samples revealed that homogenization heat treatment reduced alloy dendrites and alleviated stress concentration; after hot rolling, a large number of fragmented subgrains appeared at grain boundaries. The presence of subgrains improved alloy hardness and strength to a certain extent, achieving a favorable combination of strength and toughness.

**Figure 3:**
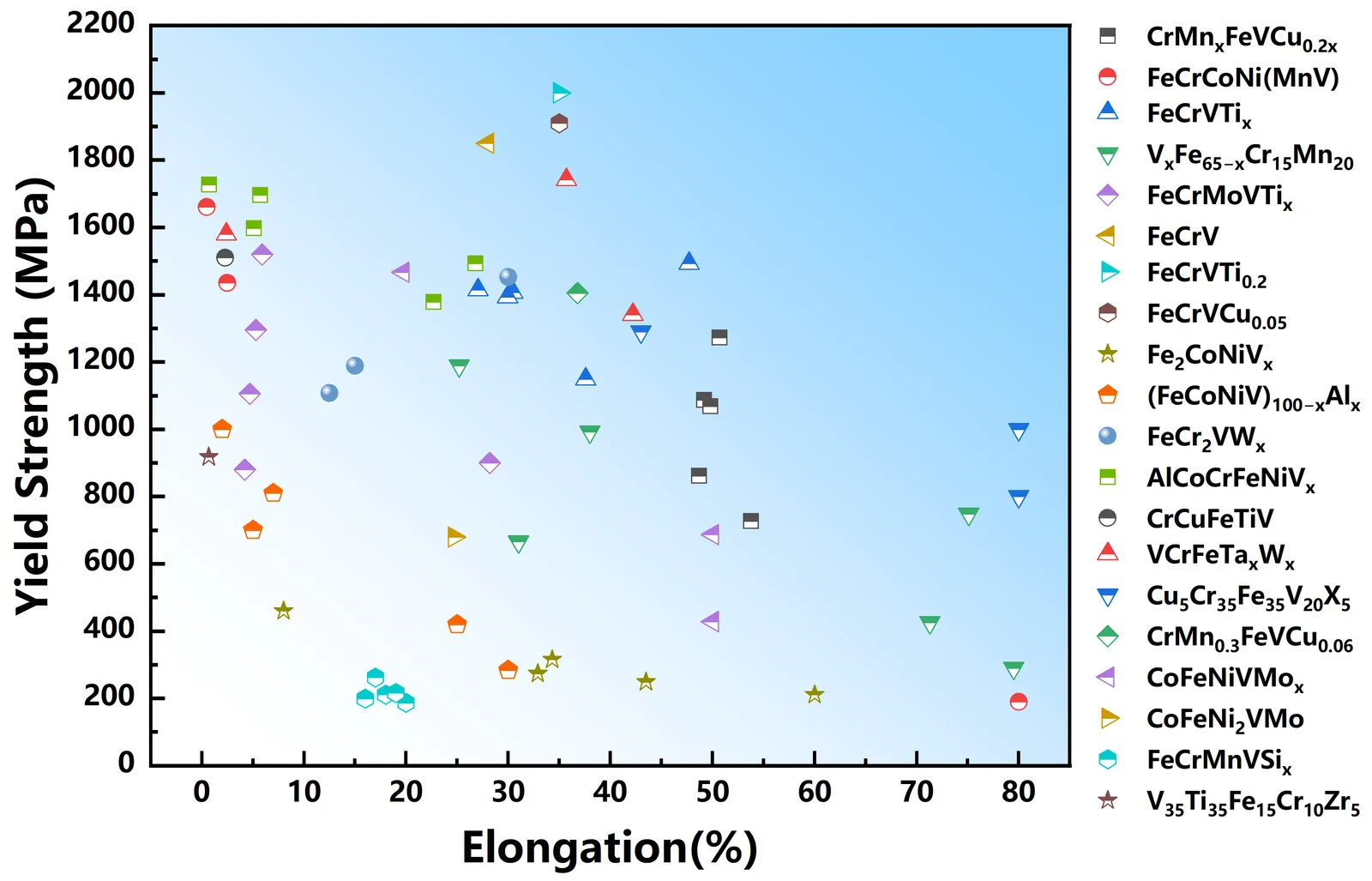
Compressive mechanical properties of Fe-V-based HEAs at room temperature.

The microstructure of HEAs directly determines the mechanical properties of the material. Microstructural characteristics include phase composition, grain size, dislocation density, twins, stacking faults, and the size and distribution of precipitated phases. All these microstructural features, whether acting alone or synergistically, have a significant impact on the strength and toughness of HEAs. It is impossible to effectively classify and discuss the diverse microstructures based solely on existing research results. Since most Fe-V-based HEAs are composed of a single solid solution or a solid solution matrix with intermetallic compound second phases, they are simply classified here into FCC-phase HEAs, BCC-phase HEAs, and multi-phase HEAs. Combining Figure 3, it is not difficult to see that although the number of Fe-V-based HEAs with a single FCC phase is limited, they are mostly concentrated in the lower right part of the graph, i.e., the alloys exhibit low strength and high toughness. In contrast, Fe-V-based HEAs with a single BCC phase often have higher yield strength and lower toughness. However, the situation becomes more complex for multi-phase Fe-V-based HEAs. On the one hand, by optimizing the composition to regulate the balance between two disordered solid solution phases with complementary mechanical properties, a balance between alloy toughness and strength can be achieved. A typical example is the presence of BCC + FCC dual phases in alloy [145], which enables the alloy to retain high toughness while possessing higher yield strength compared to single-phase alloys. The formation of intermetallic compounds (IM), including Laves phase and σ phase, undoubtedly also has an important impact on the mechanical properties of composite materials. The design of HEAs usually aims for the target material to be a simple solid solution, and the intermetallic compounds (IM) generated during this process often cause significant damage to alloy properties [162,163]. Research results from Salishchev [146] and Liu [149] indicate that in most cases, hard and brittle intermetallic compounds not only provide limited strength improvement but also greatly impair alloy toughness. However, appropriate IM size, number density, distribution, and morphology can improve strength with minimal plasticity sacrifice, achieving a remarkable strength-plasticity balance [155].

HEAs have a huge composition design space. Although existing literature has conducted a considerable number of studies on this alloy system, it is obviously too difficult to summarize the specific influence of different elements on the alloy system from current achievements due to different element selection, composition ratios, and complex preparation and post-treatment processes. There are many factors that can affect the yield stress of the alloy. Wen et al. [164] found through the study of various strengthening mechanisms that the yield stress of the alloy can be linearly added by the effects of multiple strengthening actions:

(14) $$\begin{array}{c} \sigma_{y}=\sigma_{ss}+\sigma_{p}+\sigma_{GB}+\sigma_{TB}+\sigma_{d}+\sigma_{0} \end{array}$$

where $\sigma_{ss}$ is solid-solution strengthening, $\sigma_{p}$ is precipitate strengthening, $\sigma_{GB}$ is grain boundary strengthening, $\sigma_{TB}$ is twin boundary strengthening, $\sigma_{d}$ is dislocation strengthening, $\sigma_{0}$ is lattice friction.

First is solid solution strengthening ($\sigma_{ss}$). As the most widely existing strengthening mechanism in alloys, solid solution strengthening mainly comes from the atomic size difference between different elements in the solid solution. The larger the atomic size difference, the more severe the lattice distortion of the material, and the increased internal stress caused thereby will hinder dislocation movement, thereby improving the mechanical properties of the alloy. For dilute solutions, the formula of $\sigma_{ss}$ is defined as [165]

(15) $$\begin{array}{c} \sigma_{ss}=MG\left(\frac{\varepsilon_{ss}^{\frac{3}{2}}}{700}\right)c^{\frac{1}{2}} \end{array}$$

where M is the average orientation factor of the matrix (2.76 for BCC; 3.06 for FCC); G is the effective solvent matrix shear modulus, which can be calculated by the linear mixing rule ($G=\sum_{i=1}^{n}(G_{i})c_{i}$), where $G_{i}$ is the shear modulus of the *i*-th component, $c_{i}$ is the atomic percentage of the *i*-th component, c is the solute atom concentration, and $\varepsilon_{ss}$ can be calculated by the following formula:

(16a) $$\begin{array}{c} \varepsilon_{ss}=\left|\varepsilon_{G}^{\prime }-\beta \varepsilon_{b}\right| \end{array}$$

(16b) $$\begin{array}{c} \varepsilon_{G}^{\prime }=\frac{\varepsilon_{G}}{1+\frac{1}{2}\left|\varepsilon_{G}\right|} \end{array}$$

(16c) $$\begin{array}{c} \varepsilon_{G}=\frac{1}{G}\frac{dG}{dc} \end{array}$$

(16d) $$\begin{array}{c} \varepsilon_{b}=\frac{1}{a}\frac{da}{dc} \end{array}$$

In the formula, β is 3, and a is the lattice parameter of the matrix [166]. It should be noted here that c in the formula should be the atomic concentration of the element in the solid solution, and does not include the precipitated second phase.

Xu et al. [144] calculated the solid solution strengthening effect of FeCrVTi_0.2_ multi-component alloys obtained by arc melting and mechanical alloying according to this formula, and compared the obtained results with other strengthening effects. Their team found that the influence of solid solution strengthening in the two alloys only accounts for 3.9% and 3.4% of the total strengthening strength. For this result, they considered that, on the one hand, the atomic radius change of Fe, Cr, and V elements in the alloy is small, resulting in an insignificant solid solution strengthening effect; on the other hand, the addition of a small amount of Ti and Cu mainly occurs in the form of precipitated phases, so the effect on solid solution strengthening is small. Similar results have been reported in many literatures [31,167,168].

It is not difficult to see from Formula (11) and its definition that this model is mainly applicable to binary solid solution systems, while in HEAs, the proportions of various elements are often similar, and the traditional concept-based solid solution model composed of “solute” and “solvent” may not be applicable to complex element alloys including medium-entropy alloys. Wang et al. [169] proposed a modified model to calculate $\sigma_{ss}$ of BCC phase refractory HEAs on the basis of the traditional Labusch strengthening theory [170], assuming that a single BCC phase is a homogeneous solid solution:

(17) $$\begin{array}{c} \sigma_{ss}=\frac{G}{45}{\left(\sum_{i}^{n}\varepsilon_{i}^{2}c_{i}\right)}^{\frac{2}{3}} \end{array}$$

where $\varepsilon_{i}$ is the hardening coefficient of the *i*-th component, which is related to the size and modulus mismatch of elements and the interaction force between solute atoms. This model treats the HEA solid solution as the superposition of two terminal cases: one is an undistorted lattice, and the other is a distorted lattice generated by the size and modulus mismatch between atomic species.

Second, the precipitation of secondary phases can hinder dislocation movement and also produce a strengthening effect (*σ_p_*). According to the different interaction modes between dislocations and precipitated phases, the precipitation strengthening mechanism can be divided into two different types. The first is the Orowan bypass mechanism, and the second is the shearing mechanism [164]. Usually, when the precipitated particles are small enough and maintain consistency with the matrix, dislocations can cut through the particles and continue to move, and the shearing mechanism dominates. On the contrary, when the size of the precipitated particles hinders coherence with the matrix, the Orowan dislocation-precipitation bypass mechanism tends to occur [165]. The shearing mechanism is mainly applicable to small coherent phases, where dislocations will directly cut through the precipitated phases and continue to move; when the radius of the coherent phase exceeds the critical value or is an incoherent phase, dislocations tend to bypass the precipitated phases, and the strengthening effect transitions to the Orowan bypass mechanism. For the shearing mechanism, the increase in yield strength is contributed by coherent strengthening ($\sigma_{cs}$), modulus mismatch strengthening ($\sigma_{ms}$), and order strengthening ($\sigma_{os}$) [171]. The first two occur before dislocations shear the precipitated phases, and the latter two occur during the shearing of precipitated phases by dislocations. Therefore, the total strength increment of shearing precipitated phases is the larger one of $\sigma_{cs}$ + $\sigma_{ms}$ or $\sigma_{os}$ [172,173].

When bypassing the precipitated phase through the Orowan bypass mechanism, the formula for its contribution to yield strength is [174,175]:

(18a) $$\begin{array}{c} \sigma_{or}=M\frac{0.4Gb}{\pi \lambda }\frac{ln(2\bar{r}/b)}{\sqrt{1-v}} \end{array}$$

(18b) $$\begin{array}{c} \lambda =\left[{\left(\frac{2\pi }{3f}\right)}^{\frac{1}{2}}-\sqrt{8/3}\right]r \end{array}$$

Here b is the size of the Burgers vector of the matrix, v is Poisson’s ratio, r is the average precipitate radius, and f is the volume fraction of precipitates.

Common precipitated phases in Fe-V-based HEAs include σ phase, Laves phase of HCP, or FCC Cu-rich and Ti-rich phases [22]. Their main effective strengthening mechanism is the Orowan bypass mechanism [176,177]. As mentioned earlier, strategically introducing suitable precipitates into Fe-V-based HEAs and adjusting their size, morphology, and distribution is an effective method to optimize the strength-plasticity balance.

Third, grain boundary strengthening ($\sigma_{GB}$) is also an important way of material strengthening. The contribution of grain boundary strengthening can usually be estimated by the Hall-Petch equation [165]:

(19) $$\begin{array}{c} \sigma_{GB}=K_{HP}d^{-\frac{1}{2}} \end{array}$$

where $K_{HP}$ is the Hall-Petch coefficient, and d is the average grain diameter. Combined with the literature, Ma et al. [19] prepared a series of low-activation V-Fe-Cr-Mn HEAs by arc melting. When the V content x increases from 0 to 50, the microhardness of V_x_Fe_65−x_Cr_15_Mn_20_ HEAs increases from 214.0 ± 7.2 HV to 528.4 ± 10.8 HV. However, further increasing the V content x to 65, the microhardness decreases significantly to 319.6 ± 27.5 HV. The yield strength of the alloy is strongly correlated with the grain size. Under the premise of keeping the phase composition basically unchanged, the grain boundary strengthening caused by the grain size plays an important role in the mechanical performance of this series of alloys.

Fourth, the influence of dislocations on the mechanical behavior of materials can be analyzed from two different angles. On the one hand, the accumulation of dislocations may form cracks in the alloy matrix, thereby reducing the plasticity and toughness of the material. On the other hand, the displacement of dislocations at the crack tip can alleviate stress concentration, thereby improving the toughness of the alloy. The Bailey–Hirsch relationship can be used for quantitative analysis of dislocation strengthening ($\sigma_{d}$) [166,178]:

(20a) $$\begin{array}{c} \sigma_{d}=M\alpha Gb\rho^{\frac{1}{2}} \end{array}$$

where α is a constant: 0.4 for BCC metals; 0.2 for FCC metals [165]. ρ is the dislocation density, which can be estimated by the Williamson–Hall expression [179,180]:

(20b) $$\begin{array}{c} \rho =\frac{2\sqrt{3}\varepsilon }{db} \end{array}$$

(20c) $$\begin{array}{c} B\cos\theta_{B}=\frac{K\lambda }{d}+4\varepsilon \sin\theta_{B} \end{array}$$

where K = 0.9; λ = 0.154 nm is the Cu Kα X-ray wavelength; d is the grain diameter; B is the true XRD peak broadening, $\theta_{B}$ is the Bragg angle; ε is the microstrain, which can be obtained by linear fitting of Formula (20c).

Cheng et al. [167] proved through calculation and analysis that the contribution of dislocations to the yield strength of FeCrVTi_x_ alloys prepared by AM is not significant. On the contrary, dislocations play an obvious role in the yield stress of FeCrV, FeCrVTi_0.2_, and FeCrVCu_0.05_ alloys prepared by mechanical alloying and spark plasma sintering [143,144]. This difference is attributed to the intense mechanical processing during mechanical alloying, where metal powders undergo continuous collision, fracture, and cold welding, resulting in a high density of dislocations.

Fifth, due to the obvious lattice distortion in HEAs, the interaction between dislocations and distorted lattices is uniformly distributed throughout the crystal. This means that moving dislocations encounter consistent frictional resistance when passing through the distorted lattice, similar to the lattice friction stress encountered in traditional alloys [168,181,182]. Among them, the lattice friction of the alloy is estimated by calculating the arithmetic average of the lattice friction of the constituent elements [183]. Therefore, the friction stress ($\sigma_{0}$) can be calculated by Formula (21):

(21) $$\begin{array}{c} \sigma_{0}=\sum_{i=1}^{n}{\left(\sigma_{0}\right)}_{i}c_{i} \end{array}$$

Formula (21) treats the lattice friction stress as a composition-weighted arithmetic average of the elemental values. This neglects the non-linear atomic interactions and the severe lattice distortion specific to chemically complex HEAs, so it provides only a first-order estimate for Fe-V-based alloys and should be treated accordingly. where the $\sigma_{0}$ data of common elements are listed in the Table 3 [184].

Sixth, coherent twin grain boundaries have a strengthening effect similar to grain boundaries. The definition of twin boundary strengthening ($\sigma_{TB}$) is similar to the Hall–Petch formula. Since twins are not common in HEAs and the strengthening effect contributes little to the yield stress calculation, only its definition formula is given in this paper [165]:

(22) $$\begin{array}{c} \sigma_{TB}=K^{TB}\lambda_{TB}^{-\frac{1}{2}} \end{array}$$

In the formula, $K^{TB}$ is a constant, approximately the same as $K_{HP}$ [185,186,187], and $\lambda_{TB}$ is the average twin boundary spacing (twin thickness).

The relevant calculation methods for yield strength are given above. At present, the theoretical formulas for toughness calculation remain unclear, but research on strengthening mechanisms indicates that appropriately increasing dislocation density and refining grain size are effective means to achieve a favorable combination of strength and plasticity. For precipitation strengthening, since the second phase is often a phase with poor mechanical properties, such as intermetallic compounds that are undesirable in high-entropy alloys, it is necessary to comprehensively control the size, quantity, density, shape, and distribution of precipitates to balance strength and toughness.

## 5. High-Temperature Properties

Advanced reactors will face more severe operating conditions, including higher operating temperatures, which also impose certain requirements on the thermal stability and high-temperature mechanical properties of reactor materials. The thermal stability of Fe-V-based multi-component alloys is usually evaluated through aging treatment. Comparing the changes in microstructure and properties of the material before and after different aging conditions, it is an important criterion for assessing the thermal stability of the material. Table 4 summarizes the data of aging treatments for Fe-V-based multi-component alloys.

**Figure 4:**
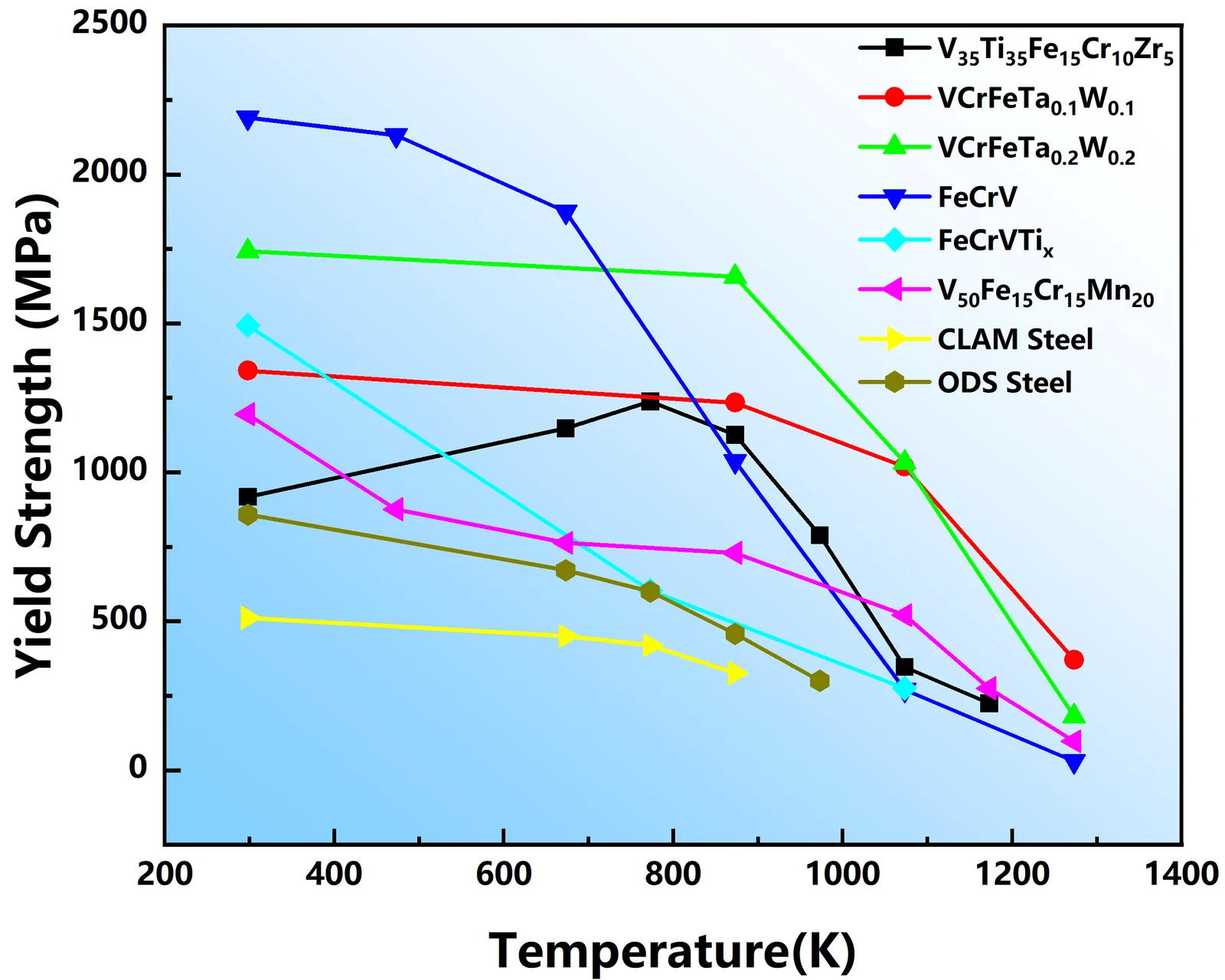
Comparison of yield strength of Fe-V-based HEAs and low-activation alloys from 298 K to 1273 K.

For the majority of Fe-V-based multi-component alloys, increasing the temperature will reduce the yield strength of the material. Therefore, the material’s resistance to thermal softening, that is, the rate at which strength decreases with increasing temperature, is the most concerning issue in high-temperature mechanical properties. The changes in strength and ductility of several typical multi-component alloys with temperature are shown in Figure 4. Although all multi-component alloys are affected by strength reduction at high temperatures, the reduction rates vary. The differences in high-temperature mechanical properties of Fe-V-based multi-component alloys are, on the one hand, related to the elements used in the alloy, and on the other hand, closely related to the heat treatment process of the alloy.

Compared with conventional reduced-activation ferritic–martensitic (RAFM) steels, which soften rapidly above roughly 550 °C, and with vanadium alloys, which retain strength to higher temperatures but suffer low-temperature irradiation embrittlement and poor oxidation resistance, the Fe-V-based HEAs reviewed here show a wider tunable window of high-temperature strength. For example, the VxFe65-xCr15Mn20 series retains a yield strength of 520 MPa at 800 °C, exceeding typical low-activation steels. However, aging experiments alone provide an incomplete picture of long-term reactor performance. They do not capture creep and fatigue behaviour, which are governed by time-dependent deformation mechanisms absent from static aging, nor oxidation and corrosion, which are strongly influenced by the coolant chemistry and the radiation field. Furthermore, irradiation-assisted microstructural evolution, including radiation-induced precipitation and segregation, can accelerate or reverse the coarsening trends observed in thermal aging alone.

## 6. Irradiation Performance

High-entropy alloys exhibit excellent irradiation resistance due to characteristics such as compositional complexity, severe lattice distortion, and the sluggish diffusion effect. Since structural materials in nuclear fusion reactors are exposed to intense neutron irradiation, the neutron irradiation resistance of low-activation high-entropy alloys has become a key focus of research. However, studies on neutron irradiation of high-entropy alloys are limited [188,189], primarily due to the scarcity of neutron irradiation resources and associated high costs. As an alternative, electron and ion irradiation are commonly used to investigate irradiation damage. Extensive academic progress has been made in understanding the irradiation resistance of high-entropy alloys [190,191,192,193,194,195,196,197], among these, Fe-V high-entropy alloys possess unique geometric and physicochemical properties that enable good compatibility with various low-activation elements, granting them inherent advantages for reactor applications.

### 6.1. Irradiation Effects of Fe-V-Based High-Entropy Alloys with FCC Structure

Due to their unique topological geometry, Fe-V-based alloys are often difficult to fabricate as simple solid solutions dominated by the FCC phase, limiting research on the irradiation damage effects of FCC-structured Fe-V-based high-entropy alloys. Current research mainly focuses on structural stability, defect morphology observation, and mechanical properties of samples before and after irradiation [198]. Wang et al. [199] prepared two single-FCC-phase high-entropy alloys (FeMnNiCr and FeMnNiCrV) and irradiated them with Au^+^ (2 MeV, 3 × 10^16^ ions/cm^2^) at 300 °C. The results showed both alloys maintained good phase stability, with no secondary phases observed post-irradiation; additionally, FeMnNiCrV contained abundant stacking faults, and exhibited lower dislocation loop number density, smaller loop size, and a lower hardening rate than FeMnNiCr after irradiation. The team attributed the superior irradiation resistance of the V-containing alloy to V-induced reduction in stacking fault energy: lower stacking fault energy promotes the formation of numerous stacking faults, which efficiently trap irradiation-induced point defects, reducing point defect concentration and further decreasing the size and density of irradiation-induced dislocation loops.

Su et al. [200] prepared Al_0.5_Cr_0.9_FeNi_2.5_V_0.2_ via arc melting, and obtained two high-entropy alloys with different L1_2_ structure contents through cold rolling and heat treatment. The alloys were irradiated with He^+^ (1 MeV, 5 × 10^16^ ions/cm^2^) at 650 °C. Results showed the L1_2_ structure (composed of Ni_3_Al) was widely distributed in the alloy, maintaining high compositional continuity with the FCC matrix and exhibiting extremely low mismatch at the coherent interface. Alloys with higher L1_2_ content exhibited smaller dislocation loop sizes, lower irradiation hardening rates, higher dislocation loop density, and a 65% reduction in swelling rate compared to those with low L1_2_ content. The team proposed two key roles of the L1_2_ structure in enhancing irradiation resistance of Al_0.5_Cr_0.9_FeNi_2.5_V_0.2_: (1) The low-mismatch coherent L1_2_ interface undergoes order-disorder transitions—high-energy ion bombardment disrupts ordered nanostructures via cascade collisions (causing disordering or dissolution), while the resulting interstitials and vacancies enhance elemental diffusion, facilitating nanostructure rearrangement/reprecipitation, which reduces early-stage point defect concentration and inhibits defect nucleation/growth; (2) The ordered-disordered L1_2_ nanostructures act as strong dispersion barriers and generate anti-phase boundaries, impeding defect cluster migration and slowing defect/He bubble growth. These results confirm that judicious introduction of secondary-phase particles into single-phase high-entropy alloys is a viable strategy to improve irradiation resistance.

### 6.2. Irradiation Effects of Fe-V-Based High-Entropy Alloys with BCC Structure

Compared to FCC high-entropy alloys, BCC high-entropy alloys typically exhibit more severe lattice distortion, higher stacking fault energy, and distinct defect microstructures, leading to different irradiation responses [201,202]. Studies demonstrate that BCC high-entropy alloys possess lower defect generation rates [203], lower irradiation hardening rates [204], and a stronger ability to inhibit He bubble nucleation/growth than FCC alloys, endowing them with unique advantages in resisting irradiation damage.

Li et al. [143] prepared FeCrV multi-principal element alloys via powder metallurgy and irradiated them with Au^2+^ (2 MeV, 160 dpa) at 300 °C. Results showed FeCrV had an irradiation hardening rate of 40%, far lower than pure iron (212%) under identical conditions. As a typical low-activation BCC high-entropy alloy, FeCrV has garnered increasing attention in recent years. Su et al. [205] investigated the effect of Al addition on FeCrV’s irradiation resistance: two equiatomic alloys (FeCrV and AlFeCrV) were prepared via arc melting and irradiated with Au^2+^ (6 MeV, 1.5 × 10^16^ ions/cm^2^) at 500 °C. Irradiation induced obvious voids in FeCrV, while no voids were observed in AlFeCrV, indicating excellent irradiation swelling resistance. The team attributed this to Al-induced increases in lattice distortion and chemical complexity, which altered the electronic system of the alloy, reduced electrical conductivity and thermal conductivity, prolonged irradiation energy dissipation, eliminated more defects, and reduced cumulative damage. Additionally, AlFeCrV exhibited a softening effect (~1.15%) in hardness tests; this was explained by the alloy’s low melting point, which caused significant annealing during high-temperature irradiation—an effect that outweighed irradiation-induced hardening, leading to overall softening.

Studies show single-phase and dual-phase BCC high-entropy alloys better inhibit He bubble nucleation and growth than FCC alloys [206,207,208]. This is due to: (1) the lower packing density of BCC than FCC structures, and the interface between the second or precipitated phase and matrix in dual-phase alloys acting as He atom traps, enabling BCC/dual-phase alloys to accommodate more He atoms [203]; (2) severe lattice distortion in BCC alloys further hindering He atom diffusion. Cheng et al. [209] designed FeCrVTi_x_ (x = 0, 0.1, 0.3) by adjusting Ti content. Ti at room temperature mainly exhibits an HCP structure, so minor LAVES phases formed post-arc melting and homogenization. They investigated He bubble evolution under varying irradiation doses, temperatures, and increasing Ti dose; He bubble diameter in the He peak region increased, while number density first rose then fell; under identical irradiation, the BCC phase outperformed the LAVES phase in resisting He bubble growth. This phenomenon has been widely observed in many types of alloys. On the one hand, this is attributed to the increase in irradiation dose, which increases the helium concentration in the alloy and increases the number of helium atoms for the nucleation of helium bubbles; on the other hand, the increase in defect concentration provides more nucleation sites for helium bubbles. These factors promote the nucleation and growth of helium bubbles, causing an increase in the number density and size of helium bubbles in the alloy. When the helium bubbles grow to a certain extent, they merge, resulting in a decrease in helium bubble density at high doses.

The radiation damage results of the alloy under different irradiation temperatures show that high temperature greatly promotes the nucleation and growth of helium bubbles in the alloy. Regarding the influence of irradiation temperature on the evolution of helium bubbles, it is proposed to determine the evolution mechanism of helium bubbles in these materials through the ratio of the diffusion coefficient (R_He/V_) of helium atoms and the diffusion coefficient of vacancies. (1) When RHe/V > 10^6^, He diffusion is dominated by lattice atom collision displacement; vacancies are immobile. (2) When 10^2^ < RHe/V < 10^6^, the vacancy diffusion coefficient increases sharply with temperature; vacancy diffusion must be considered in bubble evolution, but the He diffusion coefficient remains higher than the vacancy diffusion coefficient. He diffusion occurs via non-thermal substitution by self-interstitial atoms; that is, He atoms are displaced from vacancies by self-interstitials and recaptured by other vacancies. (3) When RHe/V < 10^2^, He diffusion is vacancy-dominated; He atoms are trapped by thermal vacancies and transition between vacancies. Notably, models (1) and (2) are idealized and do not account for thermal vacancies generated by thermal activation.

BCC-structured Fe-V also exhibits excellent resistance to irradiation-induced segregation and phase transformation. Dias et al. [210] irradiated BCC + FCC dual-phase CuCrFeTiV high-entropy alloys with Ar^+^ (300 keV, 3 × 10^17^ ions/cm^−2^); no obvious structural or morphological changes were observed in the irradiated area of the alloy. CuCrFeTiV high-entropy alloys exhibit excellent structural and compositional stability. Similarly, Ye et al. [211] prepared multi-phase Ti_8_V_22.5_Cr_22.5_Mn_22.5_Fe_22.5_Si_2_ high-entropy alloys and used Si^4+^ at 500 keV to conduct irradiation experiments up to 188 dpa at 450 °C; their experimental results show that the alloy not only has strong structural stability but also exhibits a certain degree of lattice shrinkage. The team explains this phenomenon as the mismatch interface between the precipitation phase and the matrix can serve as an effective trap for point defects, capturing silicon atoms introduced by irradiation and mitigating lattice distortion.

At present, most research on low-activation high-entropy alloys focuses on the relationship between composition design, microstructure and mechanical properties. There are relatively few studies on high-temperature mechanical properties and radiation resistance. Due to low-activation requirements for reactor-grade Fe-V-based alloys, the selection of elements is constrained. Maximizing BCC phase formation is prioritized to control He bubble nucleation, He bubble growth and enhance irradiation resistance. However, the high strength and low toughness of the BCC phase itself impose certain limitations on its processing and application. To address this, scholars propose introducing high-density nano-precipitates or dispersed oxides into the BCC matrix [198]. These uniformly distributed second phases not only improve mechanical properties but also trap irradiation-induced point defects, reducing matrix defect concentration and enhancing irradiation resistance.

Beyond alloy design and development, improving irradiation experiments and conducting in-depth irradiation damage analysis are urgent needs for Fe-V-based alloys. Current irradiation experiments rely mostly on single-particle irradiation, while fusion reactors and other advanced reactors face simultaneous irradiation by H, He and heavy ions; the synergistic effects of multiple particles cause more severe damage [212,213]. Research on multi-particle synergistic effects mainly used single-beam sequential irradiation or multi-beam simultaneous irradiation, and it has yielded preliminary results. Irradiation-induced damage is diverse; research focuses on phase stability, defect characterization, swelling resistance, and irradiation hardening, while studies on ductile-brittle transition temperature, impact toughness, tensile properties, and irradiation creep are limited by experimental conditions. Thus, comprehensive irradiation damage evaluation of Fe-V-based alloys under reactor-like conditions, combined with rigorous system simulation, is essential to fully understand their potential in advanced reactors.

The irradiation data compiled in Table 5 are almost exclusively from ion irradiation that limits their direct comparability. The multi-beam and sequential-irradiation studies summarized in Table 5 are a first step toward the synergistic H–He–heavy-ion conditions relevant to fusion, but full validation still requires neutron-irradiation campaigns.

**Figure 5:**
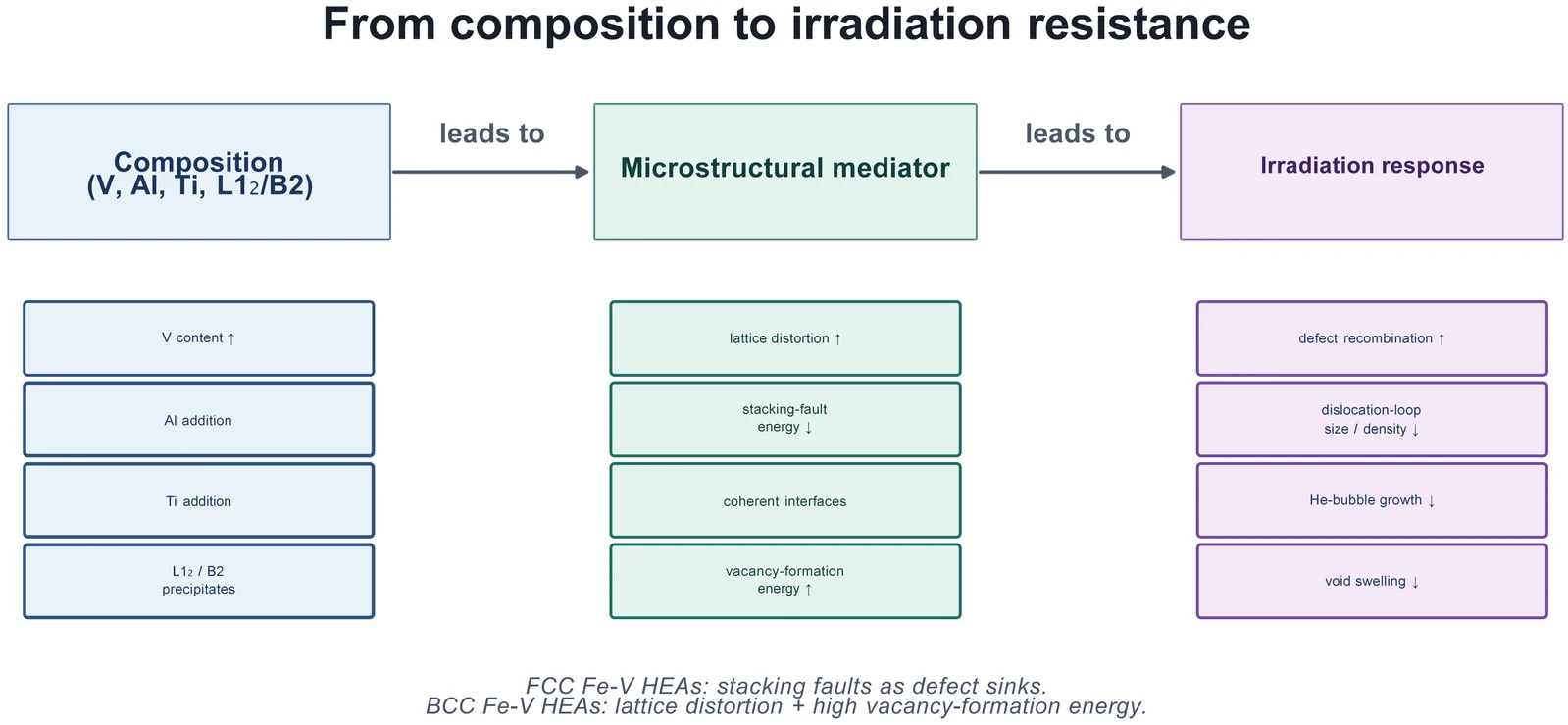
From composition to irradiation resistance of Fe-V-based HEAs. Compositional variables (V, Al, Ti, L1_2_/B2) act through microstructural mediators (lattice distortion, stacking-fault energy, coherent interfaces and vacancy-formation energy) to control defect recombination, dislocation-loop size and density, helium-bubble growth and void swelling.

Across the FCC and BCC studies reviewed above, a consistent picture emerges for the role of V. In FCC Fe-Mn-Ni-Cr alloys, V lowers the stacking-fault energy, promoting the formation of abundant stacking faults that act as sinks for irradiation-induced point defects, which reduces the point-defect concentration and thereby the size and density of dislocation loops. In BCC alloys, V contributes to severe lattice distortion and chemical complexity, which reduce defect mobility and promote defect recombination, delaying the nucleation and growth of defect clusters. At the vacancy level, the high vacancy-formation energy of BCC multi-component V-containing alloys inhibits the absorption of vacancies by helium bubbles, directly suppressing bubble growth and void swelling. Figure 5 shows the relationship between compositional variation and microstructural mediator as well as irradiation response.

Compared with RAFM steels, which suffer pronounced irradiation hardening, He embrittlement and swelling above intermediate doses, the FeCrV family shows markedly lower irradiation hardening (40% for FeCrV versus 212% for α-Fe under identical Au^2+^ irradiation) and, with Al or L1_2_ additions, greatly reduced swelling. Compared with vanadium alloys, which share the low-activation and high-temperature advantages but suffer low-temperature irradiation embrittlement and rapid oxidation, Fe-V-based HEAs offer compositional flexibility that allows the BCC matrix to be combined with ductile FCC or coherent L1_2_ second phases to recover toughness without sacrificing swelling resistance. Irradiation tolerance in Fe-V-based HEAs arises from a combination of high defect-sink density, severe lattice distortion, and coherent interfaces, which together reduce the surviving point-defect population that would otherwise drive loop growth, hardening, and swelling. Because the studies use different ion species, energies, and temperatures, the magnitudes are not directly comparable, but the underlying mechanism is consistent.

## 7. Summary

This research reviews the composition design, preparation methods, room-temperature mechanical properties, high-temperature properties, and the focused anti-radiation properties of Fe-V-based HEAs.

The composition design space of high-entropy alloys is vast. In the past two decades, studies on the design strategies of high-entropy alloys have given rise to a series of empirical criteria based on the Hume-Rothery principle and thermodynamic parameters. The use of these criteria can simply, economically, and quickly determine whether the designed high-entropy alloy system can have the target microstructure. However, the creation methods of these empirical criteria are destined to have significant limitations, so the results of different criteria often contradict each other, and the evaluation of the effectiveness of the criteria is also an important part of the design of Fe-V-based HEAs.

The complex interaction among multiple components of Fe-V-based HEAs has long severely hindered the application of simulation calculation methods in this series of alloys. However, with the development of multi-scale calculation frameworks, computer modeling technology has enabled the design of Fe-V-based HEAs to shift from the traditional “passive” prediction of alloy phase composition using empirical criteria to the active development of unexplored Fe-V-based HEA systems using general models. The comprehensive use of simulation methods such as DFT, MD, CALPHAD, and PFM provides scholars with research methods at multiple scales, from micro to macro, making it possible to analyze the interatomic interactions, phase composition, and phase evolution of Fe-V-based HEAs and the micro-metallurgical mechanism with far greater convenience than conventional experimental methods.

The preparation process is an important part of materials research. Fe-V-based HEAs inherit traditional alloy preparation methods and have derived new preparation processes. Many high-performance Fe-V-based HEAs have been designed for different application environments, including blocks, coatings, and films for surface modification, high-strength and high-toughness fibers, dense alloy powders, and newly developed additive manufacturing HEAs. Currently, the preparation of Fe-V-based HEAs is mainly based on vacuum arc melting, and other preparation methods are often limited by processing scale and cannot be used for large-scale production. In addition, research on the heat treatment of Fe-V-based HEAs is relatively limited. Developing more efficient and cheaper preparation technologies and conducting comprehensive and systematic research on alloy heat treatment are of great significance for the performance development of Fe-V-based HEAs.

Most of the tensile properties of HEAs are poor, so the mechanical property data related to them are mostly derived from compression experiments. Due to their own topological structure and physical-chemical properties, the phase composition of Fe-V-based HEAs often has a BCC matrix as the main component, and the hard and brittle BCC phase makes the originally limited toughness of the alloy even more inadequate. In addition to the processing methods mentioned earlier, selective addition of some elements is also an important means to improve the room-temperature mechanical properties of the alloy. On the one hand, soft and ductile FCC phases can be introduced into the alloy to form a dual-phase high-entropy alloy with excellent comprehensive properties; on the other hand, by adding elements with a large atomic radius difference, the lattice distortion effect of Fe-V-based HEAs can be further exacerbated, thereby enhancing the solid solution strengthening effect. However, it should be noted that elements such as Co and Ni, which are often used to increase the toughness of the alloy, have high activation characteristics; elements such as Ti and Cu, although they can increase the strength of the alloy, are prone to the formation of metal intermetallic compounds with poor mechanical properties, and the addition amount should be particularly noted. Currently, there have been considerable achievements in the experimental studies of the room-temperature mechanical properties of Fe-V-based HEAs, but the data related to creep performance, fatigue performance, ductile-brittle transition temperature, and high-temperature mechanical properties are still limited.

Fe-V-based HEAs with a BCC phase have better anti-radiation performance due to their more severe lattice distortion effect and unique electronic properties. For the application of reactors, especially fusion reactors, it is still worth considering how to replace the highly activated elements in alloys while maintaining certain mechanical properties. The research on neutron irradiation of Fe-V-based HEAs is still relatively limited. The synergistic effect of H and He, as well as the irradiation damage behavior of the alloy under high dpa, still requires further exploration. It is worth noting that the current development of Fe-V-based HEAs, whether in computational simulation or experiments, has been rather conservative in terms of element selection. Elements such as B and Be, which have significant performance impacts when added in small amounts to the alloy, are rarely involved. Several directions would accelerate the development of Fe-V-based HEAs for reactor use. The integration of CALPHAD with ML, and the use of autonomous or inverse design, can narrow the enormous composition space toward low-activation single-phase compositions while minimizing trial-and-error cost. High-throughput experimentation can validate the model predictions at a rate commensurate with the search space. Multiscale modelling that links DFT, MD, PFM and CALPHAD through machine-learned interatomic potentials would resolve the current bottlenecks of scarce potentials and immature databases.

## Figures and Tables

**Table 3:** Summary of $\sigma_{0}$ and $K_{HP}$ of pure metals.

Crystal Structure	Elements	$\sigma_{0}$ (MPa)	$K_{HP}$ (MPa·μm^1/2^)
BCC	V	150	380
	Nb	120	340
	Ta	80	760
	Cr	320	800
	Mo	270	630
	W	800	1000
	Fe	130	310
FC	Ni	80	230
	Cu	40	110
	Ag	60	100
	Au	150	80
	Al	10	90
HCP	Be	80	720
	Mg	70	90
	Ti	250	190
	Zr	240	280
	Hf	100	350
	Co	260	140
	Zn	20	220
	Cd	7	220

**Table 4:** Summary of aging studies on Fe-V-based HEAs.

Materials	Phase Composition	Heat Treatment	Results	Ref.
CoCrFeNiV	σ + FCC	1000 °C 24 h	The phase composition remained unchanged; the continuous network σ phase matrix transformed into coarse particles, which contained coarse spherical FCC-phase particles. The microhardness changes of the two alloys: CoCrFeNiV: increased from 524 HV to 587 HV; CoCrFeNiMnV: decreased from 650 HV to 636 HV.	[146]
FeCrCoNiMnV	σ + FCC			
FeCrVCu_0.05_	BCC + VxOy + Cu-rich phase	5 h 450/600/800 °C 5/15/30 h 600 °C	Formation of the FeV phase at 800 °C. Before 600 °C, the phases and mechanical properties remained stable, and the nanoscale Cu-rich phase and nanocrystals remained unchanged.	[144]
FeCrVTi_0.2_	BCC + VxOy + Ti(C,N)		Formation of the FeV phase at 800 °C; formation of the Laves phase at 600 °C.	
Cu_5_Cr_35_Fe_35_V_20_Ti_5_	BCC + Laves	700 °C 20 h	Formation of Ti-N particles. The microhardness increased from 590 HV to 630 HV.	[155]
Cu_5_Cr_35_Fe_35_V_20_Ta_5_	BCC + Fe_7_Ta_3_		The phase composition remained unchanged, but grain growth occurred. The microhardness decreased from 560 HV to 510 HV.	
Cu_5_Cr_35_Fe_35_V_20_W_5_	BCC		Formation of the σ phase. The microhardness increased from 470 HV to 520 HV.	
FeCoNiV	FCC	1100 °C 10 h	After homogenization treatment, the BCC phase in the Al_2_ and Al_5_ high-entropy alloys dissolved into the matrix (and the Al_0_ phase)	[150]
(FeCoNiV)_94_Al_6_	FCC + BCC		The microstructure of the homogenized Al_6_ high-entropy alloy was similar to that of as-cast Al_6_. Except for the transformation of the FCC phase to the L1_2_ phase, the BCC phase in the Al_6_ high-entropy alloy not only did not disappear but also transformed into the ordered B2 phase. With the volume fraction remaining unchanged, the B2 phase underwent obvious spheroidization.	
(FeCoNiV)_89_Al_11_	FCC + BCC		The BCC phase transformed into the B2 phase, and a large number of L1_2_ phases consistent with the Kurdjumov-Sachs (K-S) orientation relationship with the B2 phase precipitated.	
(FeCoNiV)_84_Al_16_	BCC		Single B2 phase	
(FeCoNiV)_80_Al_20_	BCC		Single B2 phase	

**Table 5:** Summary of irradiation damage studies on Fe-V-based HEAs.

Materials	Phase Composition	Irradiation Conditions	Research Results	Ref.
Eurofer97	FCC	300 °C 0.32 dpa	Low (reduced-activation design): W, Ta, V, Al replace Mo, Nb, Ni, significantly reducing activation.	[214]
316 SS,	FCC	400 °C 25 dpa	Marked low-temperature hardening; strength decreases at high irradiation temperature.	[215]
SiFeVCr; SiFeVCrMo	BCC + σ	Au^+^ 25 °C 5 MeV 5 × 10^15^ ions/cm^2^	Radiation-induced phase transformation observed in SiFeVCrMo.	[216]
FeMnNiCr; FeMnNiCrV	FCC	Au^+^ 300 °C 2 MeV 3 × 10^16^ ions/cm^2^	The alloy has good phase stability and no radiation-induced segregation is observed; the addition of vanadium reduces the size and number of dislocation loops, reducing the radiation hardening rate and swelling rate of the alloy.	[199]
FeCrV	BCC	Au^+^ 300 °C 2 MeV 3 × 10^16^ ions/cm^2^ 160 dpa	High density of dislocation tangles; superior resistance to radiation hardening compared to Fe.	[143]
FeCrVTi_0.2_; FeCrVCu_0.05_	BCC + V_x_O_y_ +Ti(C,N)/Cu-rich phase	Sequential ion irradiation (Fe^2+^ + H^+^)-He 450 °C Fe: 5.01 dpa H: 1484a ppm He: 800a ppm 4.7 × 10^15^/1.8 × 10^15^ ions/cm^2^	Excellent resistance to radiation hardening in nanoindentation test (hardening rate: 1.89%/1.39%).	[217]
FeCrV; FeCrVTi_0.1_; FeCrVTi_0.3_	BCC; BCC + LAVES BCC + LAVES	He^2+^ 500 °C 1.4 MeV 1 × 10^16^/5 × 10^16^/1 × 10^17^ ions/cm^2^; 25 °C/300 °C/500 °C 1.4 MeV 1 × 10^17^ ions/cm^2^	The effects of different irradiation intensities, irradiation temperatures and Ti contents on the evolution behavior and mechanism of He bubbles in this alloy are explained.	[209]
CuCrFeTiV	BCC + FCC	Ar^+^ 25 °C 300 keV 3 × 10^15^/3 × 10^16^/3 × 10^17^/3 × 10^18^ ions/cm^2^	Demonstrates the differences in the types of defects produced when alloys receive different doses of irradiation. The alloy has excellent structural stability.	[210]
		Ar^+^ 25 °C 300 keV 3 × 10^20^ ions/cm^2^	After XRD testing, it has high microstructural stability and resistance to radiation damage.	[218]
FeCrV; AlCrFeV	BCC	Au^2+^ 500 °C 6 MeV 1.5 × 10^16^ ions/cm^2^	Compared with α-Fe, the voids in CrFeV are smaller and sparser, while no voids are observed in AlCrFeV, which has better irradiation expansion performance; AlCrFeV hardness decreases after irradiation.	[205]
Al_0.5_Cr_0.9_FeNi_2.5_V_0.2_	FCC + L1_2_	He^+^ 650 °C 1 MeV 5 × 10^16^ ions/cm^2^	The L1_2_ structure in the alloy can reduce the concentration of residual defects after irradiation and the migration of defect clusters through self-healing and structural complexity, thereby improving the radiation resistance of the alloy.	[200]
V VCr V_50_Cr_15_Fe_35_ V_50_Cr_15_Fe_15_Mn_20_	BCC	He^+^ 450 °C 400 keV 5 × 10^16^ ions/cm^2^	The size of the He bubble is smallest in VCr The high vacancy formation energy of BCC structure multi-component alloys can inhibit the absorption of vacancies by He bubbles.	[219]
CrFeMoV	BCC	He^+^ 25 °C 130 keV 1000/5000/20,000 appm 1.54 × 10^15^/7.7 × 10^15^/30.8 × 10^15^ ions/cm^2^	The lattice distortion effect of high-entropy alloys destabilizes defect clusters, increases the recombination rate of residual defects, and delays the growth of defect clusters, thereby reducing the radiation hardening rate.	[220]
(CrFeTiTa)_70_W_30_	BCC + LAVES + Titanic oxide	Ar^+^/He^+^ + D^+^/ Ar^+^ + He^+^ + D^+^ 2.4 × 10^20^ ions/cm^2^	When implanted with He^+^ + D^+^, deuterium retention rate is 5.47%; when implanted with Ar^+^ + He^+^ + D^+^, deuterium retention rate of 14.41% forms a defect that acts as a trap.	[221]
VFeTiTaW	BCC + LAVES+ Titanic oxide	Ar^+^/He^+^ + D^+^/ Ar^+^ + He^+^ + D^+^ 2.4 × 10^20^ ions/cm^2^	When implanted with Ar^+^ + He^+^ + D^+^, deuterium retention rate of VFeTiTaW decreased from 13.20% to 8.58%	
Ti_8_V_22.5_Cr_22.5_Mn_22.5_Fe_22.5_Si_2_	BCC + Ti_5_Si_3_ + TiFe_2_Si	Si^4+^ 450 °C 500 keV 3.21 × 10^16^ ions/cm^2^ 3.21 × 10^16^ ions/cm^2^	The alloy has excellent phase stability. Due to the synergistic effect of the mismatch interface between the precipitate phase and the matrix, traps called point defects, combined with the inherent properties of high-entropy alloys, prevent the alloy from forming voids and lattice shrinkage under high-dose irradiation.	[211]

## Data Availability

No new data were created or analyzed in this study. Data sharing is not applicable to this article.
